# Morphological Transformation and Force Generation of Active Cytoskeletal Networks

**DOI:** 10.1371/journal.pcbi.1005277

**Published:** 2017-01-23

**Authors:** Tamara Carla Bidone, Wonyeong Jung, Daniel Maruri, Carlos Borau, Roger D. Kamm, Taeyoon Kim

**Affiliations:** 1 Department of Mechanical and Aerospace Engineering, Polytechnic University of Turin, Turin, Italy; 2 School of Mechanical Engineering, Purdue University, West Lafayette, Indiana, United States of America; 3 Weldon School of Biomedical Engineering, Purdue University, West Lafayette, Indiana, United States of America; 4 Department of Mechanical Engineering, Aragon Institute of Engineering Research (I3A), University of Zaragoza, Zaragoza, Spain; 5 Departments of Biological Engineering and Mechanical Engineering, Massachusetts Institute of Technology, Cambridge, Massachusetts, United States of America; Northeastern University, UNITED STATES

## Abstract

Cells assemble numerous types of actomyosin bundles that generate contractile forces for biological processes, such as cytokinesis and cell migration. One example of contractile bundles is a transverse arc that forms via actomyosin-driven condensation of actin filaments in the lamellipodia of migrating cells and exerts significant forces on the surrounding environments. Structural reorganization of a network into a bundle facilitated by actomyosin contractility is a physiologically relevant and biophysically interesting process. Nevertheless, it remains elusive how actin filaments are reoriented, buckled, and bundled as well as undergo tension buildup during the structural reorganization. In this study, using an agent-based computational model, we demonstrated how the interplay between the density of myosin motors and cross-linking proteins and the rigidity, initial orientation, and turnover of actin filaments regulates the morphological transformation of a cross-linked actomyosin network into a bundle and the buildup of tension occurring during the transformation.

## Author Summary

Contractile networks and bundles generate mechanical forces required for various cellular processes, particularly cell division and migration. In many of these processes, networks are structurally reorganized into bundles by the activity of molecular motors. During this morphological transformation, filaments constituting networks are reoriented and undergo deformation and turnover, and large tensile forces are generated and sustained in bundles. However, it remains inconclusive how the morphological transformation and force generation are regulated. Here, using a rigorous computational model, we quantitatively demonstrated that the interplay between several factors determines the characteristics of generated tensile force and regulates the transformation from networks to bundles. Thus, results in this study provide insights into the physical and mechanistic basis of the complex transition from networks to bundles observed in cells.

## Introduction

The actin cytoskeleton plays an important role in various cellular processes, such as changes in cell shape, cytokinesis, and cell migration [1]. Much of the mechanical forces required for these processes are generated by interactions between actin filaments (F-actin) and myosin II motors [2]. Actomyosin contractility regulates structural organization of the actin cytoskeleton and its rheological properties by interacting and competing with the dynamics of actin cross-linking proteins (ACPs) and actin filaments. For example, during *Dictyostelium* furrow ingression, interactions between myosin and ACP dynamics control cytokinesis contractility dynamics and mechanics [3]. In addition, during fission yeast cytokinetic ring assembly, an increase in ACP density prevents clump formation [4, 5]. Representative cytoskeletal structures that are regulated by actomyosin contractility are various types of bundles, such as stress fibers, random polarity bundles, cytokinetic rings, and transverse arcs [6]. Despite similarity in their structural organization, these bundles are formed via very distinct mechanisms. Dorsal stress fibers are assembled via formin-driven polymerization of actin filaments occurring outside adhesion sites. Transverse arcs, that are located at the interface between lamellipodia and lamella, form via actomyosin-driven condensation of actin filaments within the lamellipodia [7]. During the condensation, actin filaments whose barbed ends are initially biased toward the cell margin are reoriented and thus become parallel to the margin. Transverse arcs move away from the cell margin and eventually coalesce with dorsal stress fibers, to transmit contractile forces to surrounding environments, without direct attachment to focal adhesions [8].

Several aspects regarding structural reorganization of a network into a bundle have been investigated in previous numerical studies. It was shown that an increase in myosin density induces a structural transition from networks into bundles through a series of hierarchical steps [9] with enhancement of forces generated by the actomyosin structures [10]. In addition, a recent study demonstrated that an increase in ACP density above a threshold value leads to a switch-like transition from random networks to ordered, bundled structures [11]. However, owing to the highly simplified models and limited scopes of the previous studies, it still remains inconclusive how a network is transformed into a bundle, how force is generated, and what happens on actin filaments during the structural reorganization. Several biophysical factors are likely to impact network transformation into a bundle. For example, an extent to which actin filaments are cross-linked will play an important role. If filaments are loosely cross-linked, they may be reoriented relatively easily to form a bundle, but low network connectivity could be antagonistic to the stability of formed bundles and generated forces. By contrast, if actin filaments are heavily cross-linked, they may not easily rotate without significant deformation. Because of the low bending rigidity of actin filaments, myosin motor activity could result in buckling during reorientation and compaction of cross-linked actin filaments. As suggested by a previous theoretical study [12], filament buckling may play a critical role in either force generation or bundle formation or in both. In addition, fast turnover of actin filaments occurring via diverse actin binding proteins within cells has potential to modulate the morphological transformation and force generation. Using only experiments, it is challenging to accurately evaluate relative importance of each of these factors and isolate their effects.

In this work, using an agent-based computational model, we systematically investigated morphological transformation of an actomyosin network into a bundle and force generation during the transformation. We investigated effects of diverse biophysical parameters on network compaction into a bundle, which were not systematically studied in previous computational works. Specifically, we focused on the impacts of the densities of ACPs and motors and of the rigidity, initial orientation, and turnover of actin. Results from the study were discussed in the context of the assembly of transverse arcs observed in migrating cells [7]. This study provides new insights into mechanistic understanding of a role of the interplay between various biophysical factors in bundle formation and force generation.

## Results

### Model overview

**Fig 1:**
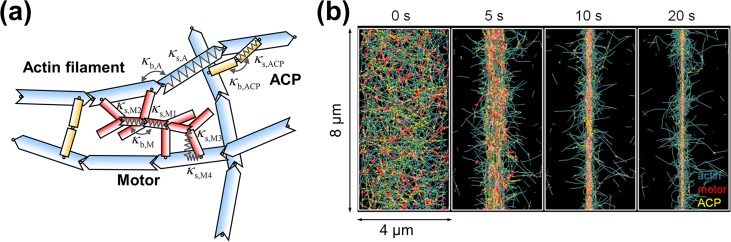
An agent-based computational model was employed for studying bundle formation. (a) Actin filaments, motors, and actin cross-linking proteins (ACPs) are simplified into cylindrical segments connected by elastic hinges. Actin filaments (blue) are modeled as a series of cylindrical segments with polarity (barbed and pointed ends). Motors (red) consist of a backbone with symmetric polarity and with arms representing myosin motor heads. ACPs (yellow) are modeled as two parallel arms. Arms of motors and ACPs can bind to actin filaments. Equilibrium lengths of segments of actin filaments (A), motors (M), and ACPs are maintained by extensional stiffness (*κ*_s_), whereas equilibrium angles between segments are maintained by bending stiffness (*κ*_b_). (b) An example of bundle formation via compaction of a network. The network (4×8×0.5 μm) consists of actin (teal) with concentration *C*_A_ = 40 μM, motor (red) with density *R*_M_ = 0.08, and ACP (yellow) with density *R*_ACP_ = 0.02. A periodic boundary condition is applied only in the y-direction. After motors start walking at *t* = 0 s, initially homogeneous network compacts into a bundle within 20 s.

We employed our previous coarse-grained Brownian dynamics model for actomyosin structures [13]. In the model, actin filaments, actin cross-linking proteins (ACPs), and motors are simplified into interconnected cylindrical segments (Fig 1A). Actin filaments consist of serially-connected cylindrical segments with polarity (barbed and pointed ends). ACPs are composed of a pair of cylindrical segments. Each motor has a backbone structure with 8 arms, each of which represents 8 myosin heads. Displacement of the segments is governed by the Langevin equation. Harmonic potentials with bending (*κ*_b_) and extensional stiffnesses (*κ*_s_) maintain equilibrium angles and lengths, respectively, formed by the segments. Repulsive forces account for volume-exclusion effects between actin filaments. Stochastic forces satisfying the fluctuation-dissipation theorem are applied to induce thermal fluctuation [14]. Positions of the segments are updated at each time step using the Euler integration scheme. ACPs bind to actin filaments at a constant rate and also unbind from actin filaments in a force-dependent manner following Bell’s equation [15]. A motor arm binds to an actin filament and walks toward the barbed end of the actin filament, generating tensile forces. Actin undergoes nucleation, polymerization, and depolymerization, staying in either monomeric or filamentous state. We simulate treadmilling of actin filaments by imposing equal polymerization and depolymerization rates at barbed and pointed ends, respectively. To alter the treadmilling rate without a large change in average length of actin filaments, a nucleation rate is dynamically adjusted. Monomeric actin and free ACP and motor that are not bound to any actin filament are considered implicitly by their local concentrations. Self-assembly of actins, ACPs, and motors in a 3D rectangular computational domain (4×8×0.5 μm) results in a homogenous actomyosin network (Fig 1B). A periodic boundary condition is imposed in the y-direction, whereas boundaries in the x- and z-directions exert repulsive forces on the segments to keep them within the domain. After network assembly, walking of motors on actin filaments is initiated, facilitating transformation of the network to a bundle. We measured a macroscopic force generated by a bundle and also microscopic forces acting on ACPs and motors. Definitions of terms are listed in S1 Table, and detailed values of parameters are listed in S2 Table.

### Densities of motors and ACPs critically regulate bundle formation and tension generation

**Fig 2:**
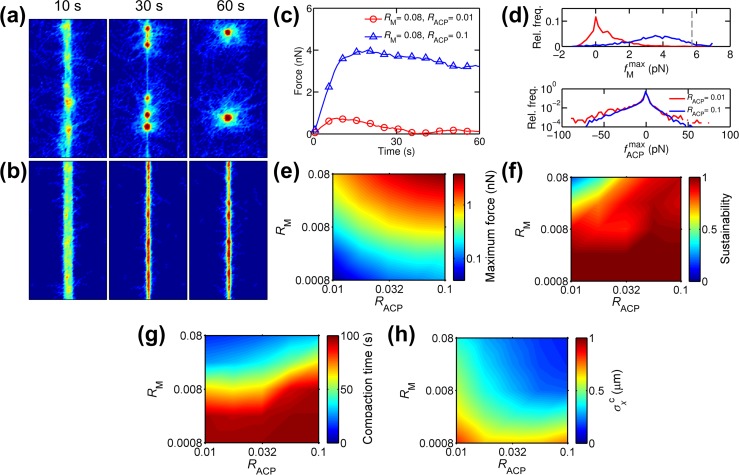
Densities of motors (*R*_M_) and ACPs (*R*_ACP_) determine characteristics of bundle formation and tension generation. (a-b) Snapshots showing actin density for (a) unsuccessful (*R*_M_ = 0.08, *R*_ACP_ = 0.01) and (b) successful bundle formation (*R*_M_ = 0.08, *R*_ACP_ = 0.1). (a) At *t* = 10 s, the bundle has heterogeneous actin distribution with dangling actin filaments. The bundle further aggregates into a few clumps over time (*t* = 30 s and 60 s). (b) The bundle has relatively homogeneous actin distribution at *t* = 10 s and remains stable during and after compaction (*t* = 30 s and 60 s). (c) Time evolution of tensile forces generated by bundles shown in (a) and (b). The case with unsuccessful bundle formation (red circle) shows lower tension which decreases faster than tension in the case with successful bundle formation (blue triangle). (d) Distribution of forces exerted on motors ($f_{M}^{\max}$) and ACPs ($f_{\mathrm{ACP}}^{\max}$) measured at peak tension for cases shown in (a) and (b). The gray dashed line indicates stall force of motors (5.7pN). The case with unsuccessful bundle formation (red) shows smaller $f_{M}^{\max}$ and larger $f_{\mathrm{ACP}}^{\max}$ than the case with successful bundle formation (blue). (e) The maximum and (f) sustainability of tensile forces generated by bundles, depending on *R*_M_ and *R*_ACP_. The sustainability ranges from 0 (not sustainable at all) to 1 (perfectly sustainable). Maximum force is positively correlated with both *R*_M_ and *R*_ACP_. Sustainability is positively correlated with *R*_ACP_ but negatively correlated with *R*_M_. (g) Compaction time as a measure of how rapidly networks transform into bundles. Networks compact faster with higher *R*_M_ and lower *R*_ACP_. (h) Standard deviation of x positions of actins at compaction time ($\sigma_{x}^{\text{c}}$) as a measure of how tightly the bundle is formed. Higher *R*_M_ and *R*_ACP_ leads to formation of tighter bundles.

Consistent with previous theoretical and experimental studies [16–18], densities of ACPs (*R*_ACP_) and motors (*R*_M_) critically affect bundle formation and tension generation. With *R*_M_ = 0.08 and *R*_ACP_ = 0.01, a homogeneous network compacted into a bundle spanning the computational domain in the y-direction within ~10 s (Fig 2A). However, the bundle was heterogeneous at 10 s in terms of actin concentration, showing a few regions with higher actin density. In addition, the bundle was highly unstable, resulting in a few separate aggregates over time. Tension measured in the bundle increased up to ~0.8 nN and then decreased to nearly zero (Fig 2C). By contrast, with *R*_M_ = 0.08 and *R*_ACP_ = 0.1, a more compact, uniform bundle was formed within 15 s, and the bundle remained intact for the duration of the simulation (Fig 2B). Tension increased up to ~4 nN, and then decreased slowly. Microscopic forces exerted on each motor ($f_{M}^{\max}$) and ACP ($f_{\mathrm{ACP}}^{\max}$) measured at maximum tension can explain the magnitude and sustainability of the generated tension (Fig 2D). Note that $f_{M}^{\max}$ and $f_{\mathrm{ACP}}^{\max}$ are positive when they are exerted toward barbed ends of actin filaments. With a large number of ACPs (*R*_M_ = 0.08 and *R*_ACP_ = 0.1), $f_{M}^{\max}$ was higher, and $f_{\mathrm{ACP}}^{\max}$ was smaller. If there are many ACPs, they share loads exerted by motors, leading to smaller force on each ACP. Since ACPs are assumed to exhibit slip-bond behavior, the smaller force on ACPs leads to less frequent unbinding events of ACPs. Thus, stable ACPs can help motors to generate higher force close to their stall force and support the force for a longer time. By contrast, with fewer ACPs (*R*_M_ = 0.08 and *R*_ACP_ = 0.01), most motors failed to attain their stall force, and each ACP supported a larger force, leading to instability of the bundle and reduction in generated tension (Fig 2D).

We systematically varied *R*_ACP_ and *R*_M_ to probe their effects on bundle formation and tension generation. Maximum tension was positively correlated with both densities (Fig 2E), whereas sustainability was proportional to *R*_ACP_ but inversely proportional to *R*_M_ (Fig 2F). We measured time evolution of standard deviation of x positions of actins (*σ*_*x*_) to quantify compaction of networks (S1 Fig). *σ*_*x*_ tends to initially decrease, indicating compaction of networks. After reaching its minimum value, *σ*_*x*_ remained constant in most cases. However, in some cases, *σ*_*x*_ increased over time, which may indicate disintegration of a bundle into aggregates. Indeed, the increase in *σ*_*x*_ occurred in cases with higher *R*_M_ and lower *R*_ACP_ where tension is not sustained well, and bundles are likely to form aggregates. In cases with very low *R*_M_, *σ*_*x*_ continuously decreased, indicating very slow compaction of networks. To quantify how fast networks compact, we defined compaction time as time at which the rate of change in *σ*_*x*_ over time becomes larger than 0.01 × (the average rate of change in *σ*_*x*_ during first 5s). The compaction time was shorter at higher *R*_M_ and lower *R*_ACP_ (Fig 2G). We used the standard deviation at compaction time ($\sigma_{x}^{\text{c}}$) as an indicator of how tightly a network is compacted in the x-direciton (Fig 2H). A tighter bundle was formed with higher *R*_M_ and *R*_ACP_. A sufficient amount of ACPs can tighten bundles by helping force generation of motors and increasing connectivity of bundles. However, ACPs slow down formation of bundles because a network becomes much more stiffer with more ACPs. In sum, a network with more motors compacted faster into a tighter bundle exerting larger tension because there are more force generators. However, the bundle and the tension are likely to be unstable, leading to bundle disintegration into aggregates and significant tension relaxation. A network with more ACPs compacted more slowly into a tighter bundle generating larger and more sustained tension.

### Buckling of actin filaments is crucial for the transformation into a bundle

**Fig 3:**
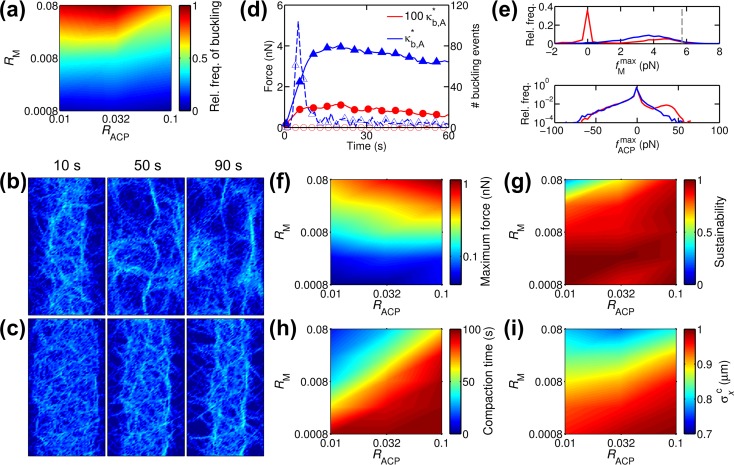
Buckling of actin filaments plays a crucial role in bundle formation and tension generation. (a) Number of actin filaments that experience buckling at least once during simulation depending on densities of motors (*R*_M_) and ACPs (*R*_ACP_), normalized by the largest number. Buckling takes place more frequently with higher *R*_M_ and lower *R*_ACP_. (b-c) Snapshots showing actin density of networks where buckling is suppressed via a 100-fold increase in bending stiffness of actin filaments (*κ*_b,A_). In both networks with (b) low (*R*_M_ = 0.08 and *R*_ACP_ = 0.01) and (c) high ACP density (*R*_M_ = 0.08 and *R*_ACP_ = 0.1), bundles were hardly formed. (d) Time evolution of generated tension (solid line) and the number of buckling events (dashed line) for cases with reference bending stiffness (blue triangle, *κ*_b,A_ = $\kappa_{b,A}^{*}$) and 100-fold higher bending stiffness (red circle, *κ*_b,A_ = 100×$\kappa_{b,A}^{*}$) at *R*_M_ = 0.08 and *R*_ACP_ = 0.1. (e) Distribution of forces exerted on motors ($f_{M}^{\max}$) and ACPs ($f_{\mathrm{ACP}}^{\max}$) measured at peak tension for cases shown in (d). The legend is shared with (d). (f) The maximum and (g) sustainability of tension measured from cases (*κ*_b,A_ = 100×$\kappa_{b,A}^{*}$) with various *R*_M_ and *R*_ACP_. Compared to cases with reference bending stiffness (Fig 2E and 2F), the networks exhibit smaller maximum tension and higher sustainability, whereas proportionality to *R*_M_ and *R*_ACP_ is maintained. (h) Compaction time. (i) Standard deviation of x positions of actins at compaction time ($\sigma_{x}^{\text{c}}$). Note that the lower limit of the color scaling is much larger than that in Fig 2H.

In our previous studies, it was shown that buckling of actin filaments is necessary for contraction of a network and for force generation in a preformed bundle [16, 19]. We quantified buckling events occurring in the simulations shown in Fig 2E–2H, by tracking the ratio of end-to-end distance to contour length of actin filaments. Since most actin filaments have multiple, transiently bound motors and ACPs, buckling takes place in various ways; some of the actin filaments experienced subsequent buckling events at multiple locations over time, and buckled filaments, at times, became straight again (S2 Fig). We determined the number of actin filaments that underwent buckling at least once in each simulation by assuming that actin filaments with a ratio of end-to-end distance to contour length smaller than 0.6 are buckled. We found that buckling occurred less frequently with higher *R*_ACP_ because the critical force above which buckling occurs becomes larger with higher *R*_ACP_ (Fig 3A); this is associated with a decrease in distance between adjacent cross-linking points on an actin filament. Although motors generate larger forces with higher *R*_ACP_ (Fig 2D), the increase in the critical force required for buckling is greater, leading to less frequent buckling events. With higher *R*_M_, buckling took place more frequently since more motors generate larger contractile forces that can induce buckling. These buckling events mostly occurred during the transformation to a bundle before tension reached its maximum, rather than after the peak tension (Fig 3D).

We tested whether buckling is required for the transformation of a network into a bundle by suppressing the filament buckling via a 100-fold increase in the bending stiffness of actin filaments (*κ*_b,A_ = 100×$\kappa_{b,A}^{*}$), where $\kappa_{b,A}^{*}$ is the reference bending stiffness. At both high and low levels of *R*_ACP_, a bundle rarely formed although some of the actin filaments formed a pseudo bundle at the center (Fig 3B and 3C). At *R*_M_ = 0.08 and *R*_ACP_ = 0.1, the developed tension in a network with 100×$\kappa_{b,A}^{*}$ was much smaller than that in a network with $\kappa_{b,A}^{*}$, and buckling rarely occurred (Fig 3D). Smaller tension for the case with 100×$\kappa_{b,A}^{*}$ can be attributed to low values of $f_{M}^{\max}$; although some values reached stall force, there was a general tendency for the forces to be smaller overall than those in the case with $\kappa_{b,A}^{*}$ (Fig 3E). Negative values of $f_{\mathrm{ACP}}^{\max}$ were also slightly smaller in magnitude for the case with 100×$\kappa_{b,A}^{*}$ since ACPs sustain lower positive $f_{M}^{\max}$ in this case. Note that negative or positive $f_{\mathrm{ACP}}^{\max}$ sustain positive or negative $f_{M}^{\max}$, respectively. Positive $f_{\mathrm{ACP}}^{\max}$ showed higher value for the case with 100×$\kappa_{b,A}^{*}$, since this case exhibits a significant amount of negative $f_{M}^{\max}$ while the case with $\kappa_{b,A}^{*}$ does not.

Due to the catch-bond nature of motors, the lower positive $f_{M}^{\max}$ makes motors stay for a shorter time on actin filaments, which corresponds to a lower duty ratio of motors. Then, motors are less able to stably generate a large amount of forces. Suppression of bundle formation and generation of lower tension observed in Fig 3B–3D might originate largely from a decrease in the duty ratio rather than an increase in *κ*_b,A_. To confirm the importance of *κ*_b,A_, we ran a simulation using motors with a much higher unbinding rate (i.e. lower duty ratio) than the motors used in the case shown in Fig 2B where a stable bundle was formed. We varied one of the mechanochemical rates in the parallel cluster model [20, 21], which leads to a decrease in the stall force from 5.7 pN to 5.3 pN and an increase in the unbinding rate from 0.049 s^-1^ to 0.49 s^-1^. As shown in S3 Fig, a bundle still formed well, and tension inside the bundle and sustainability were similar to those of the reference case shown in Fig 2B and 2C. Thus, the inhibition of bundle formation and the decrease in tension result mostly from the change in the *κ*_b,A_, not the change in the duty ratio of motors.

Maximum tension measured under various values of *R*_M_ and *R*_ACP_ with 100×$\kappa_{b,A}^{*}$ (Fig 3F) was much lower than that measured with $\kappa_{b,A}^{*}$ (Fig 2E). Dependences of sustainability and compaction time on *R*_M_ and *R*_ACP_ (Fig 3G and 3H) were similar to those in the cases with $\kappa_{b,A}^{*}$ (Fig 2F and 2G). We also measured time evolution of *σ*_*x*_ for quantification of network compaction (S4 Fig). Interestingly, in cases with lower *R*_ACP_ and higher *R*_M_, *σ*_*x*_ increased beyond its initial value after reaching the minimum. $\sigma_{x}^{\text{c}}$ was overall higher in the cases with 100×$\kappa_{b,A}^{*}$ (Fig 3I) than that in the cases with $\kappa_{b,A}^{*}$ (Fig 2H), quantitatively showing suppression of bundle formation with stiffer actin filaments. Interestingly, with more ACPs, $\sigma_{x}^{\text{c}}$ was larger, which is opposite to the observation in Fig 2H. As shown in Fig 3A, buckling occurred less frequently at higher *R*_ACP_ even with $\kappa_{b,A}^{*}$. However, since a fraction of actin filaments were still buckled, the number of buckled actin filaments is not a critical factor determining the extent of network compaction. By contrast, with 100×$\kappa_{b,A}^{*}$, most of actin filaments cannot be buckled due to a significant increase in the critical buckling force. Then, network compaction becomes very sensitive to the number of buckled actin filaments because buckling is necessary for network compaction, resulting in less network compaction with higher *R*_ACP_.

In sum, these results demonstrate that even with a sufficient number of ACPs that sustain tension and help motors reach their stall force, buckling of actin filaments is required for formation of tight bundles and generation of large tension.

### Initial orientation of actin filaments regulates bundle formation and tension generation

**Fig 4:**
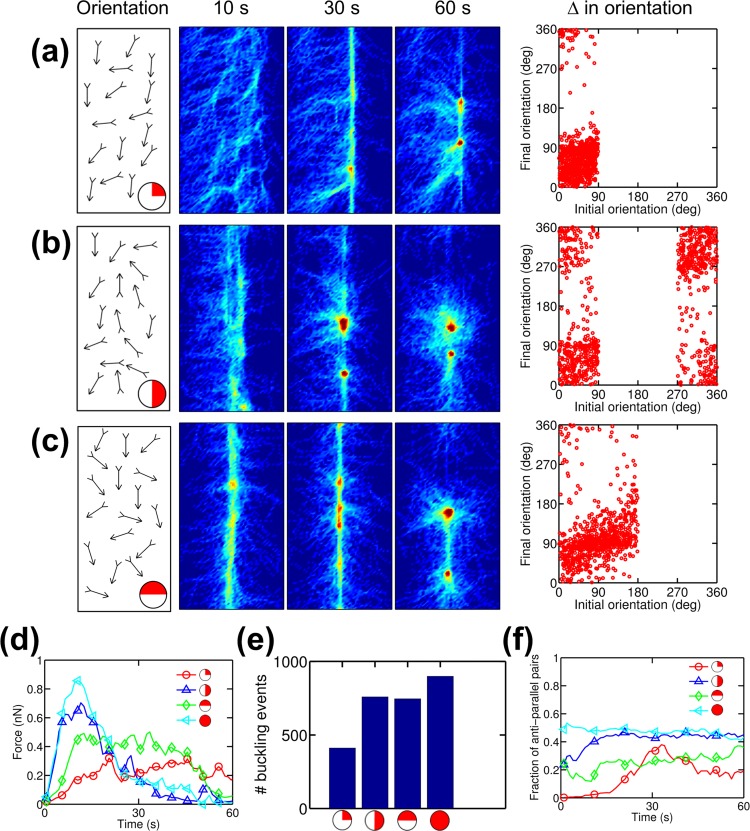
Initial orientation of actin filaments regulates bundle formation and tension generation. Densities of motors (*R*_M_) and ACPs (*R*_ACP_) used in cases shown here are 0.08 and 0.01, respectively. (a-c) (1st column) Orientations where barbed ends of actin filaments in networks are initially directed. Red on the circles located at the bottom-right corner represents the range of the orientation. Arrows in the boxes represent examples of filaments with corresponding initial orientations. (2nd, 3rd, 4th columns) Snapshots showing actin density in the networks at *t* = 10, 30, and 60 s with initial orientation indicated in the 1st column. (5th column) Initial and final orientations of actin filaments. Final orientation indicates orientation of filaments measured at a time point when compaction time is defined. (d) Time evolution of tension for cases with biased initial orientations shown in (a-c) and isotropic initial orientation. (e) Number of buckling events occurring during simulations for cases shown in (d). (f) Time evolution of a fraction of antiparallel filament pairs for cases shown in (d).

Myosin II motors compact actin filaments in lamellipodia into transverse arcs that generate contractile forces [22]. Since the barbed ends of all actin filaments in lamellipodia are directed toward the cell margin, the lamellipodia is not an isotropic actin network. We probed the effects of anisotropic initial orientations of actin filaments on bundle formation and tension generation with *R*_M_ = 0.08 and *R*_ACP_ = 0.01 by creating three networks consisting of actin filaments with biased initial orientations (Fig 4A–4C). Note that the case shown in Fig 4B where actin filaments are initially oriented toward the +x direction mimics filament orientation in lamellipodia. Compared to the reference case with isotropic orientation of filaments (Fig 2A and 2C), the networks with biased orientations showed lower maximum tension and slower bundle formation (Fig 4A–4D) because there were a smaller number of antiparallel pairs of actin filaments that are in configuration suitable for motors to produce force (Fig 4F). Interestingly, a network with barbed ends directed toward +y was effectively transformed to a bundle with significant tension despite the fact that it initially had no antiparallel pairs of actin filaments in the y-direction. We found that some of the actin filaments changed their orientations (S5 Fig and Fig 4C, right column) during network contraction (Fig 4F). Even in the network with barbed ends oriented toward +x/+y, a bundle could form slowly and generate tension due to changes in filament orientation (Fig 4A, 4D and 4F). In all cases, bundles eventually collapsed into a few aggregates; this occurred at a rate proportional to the maximum tension because larger tension accelerates destabilization of ACPs, leading to faster disintegration of bundles. We also tested the influences of initial orientation of actin filaments (diagonal or horizontal/vertical) on bundle formation and tension generated in networks, and the results overall showed similar tendencies (S6 and S7 Figs). At higher ACP density (*R*_M_ = 0.08 and *R*_ACP_ = 0.1), actin filaments tend to rotate less than those at lower *R*_ACP_ because the filaments are confined more by a larger number of ACPs (S8A, S8B and S8C Fig). However, some of the actin filaments were still able to change their orientations, contributing to tension generation (S8D and S8E Fig). Note that unlike the case with lower ACP density, the bundles were not disintegrated into aggregates, regardless of initial filament orientation. This can explain a discrepancy between the unstable bundle shown in Fig 4B formed from a network mimicking the geometry of lamellipodia and a stable bundle observed at the interface between lamellipodia and lamella. It is expected that actin filaments with numerous branching points in lamellipodia have very high connectivity between actin filaments, preventing a bundle from being disintegrated.

Taken together, these results demonstrate that networks with biased filament orientations can still be transformed to bundles owing to changes in filament orientation occurring during contraction. However, if orientations are biased, bundles are loose, and generated tension tends to be lower but is sustained for a longer time.

### Buckling is not necessary for bundle formation in networks with biased filament orientation

**Fig 5:**
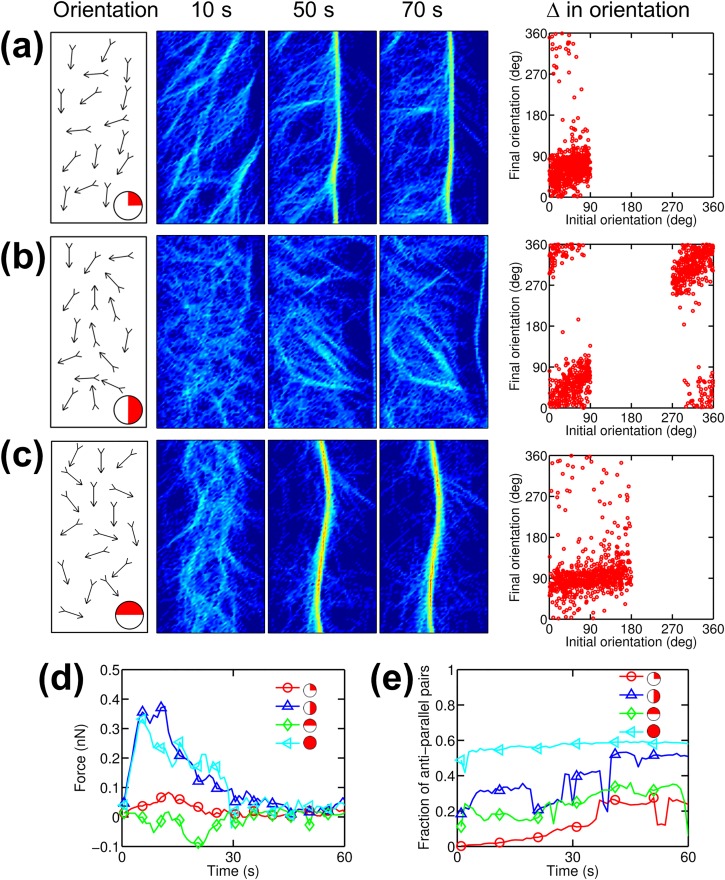
In networks with biased filament orientation, parallel filament pairs can form bundles without buckling of filaments, whereas antiparallel pairs cannot. Densities of motors (*R*_M_) and ACPs (*R*_ACP_) are 0.08 and 0.01, respectively as in Fig 4, but bending stiffness of actin filaments is increased 100-fold (*κ*_b,A_ = 100×$\kappa_{b,A}^{*}$). (a-c) (1st column) Initial orientations of actin filaments in networks. (2nd, 3rd, 4th columns) Snapshots showing actin density in the networks with initial filament orientation indicated in the 1st column. (5th column) Initial and final orientation of actin filaments. (d) Time evolution of tensile forces generated by bundles for cases with biased initial orientations shown in (a-c) and isotropic initial orientation. (e) Time evolution of a fraction of antiparallel filament pairs for cases shown in (d).

We have observed that buckling is necessary for bundle formation in networks with isotropic filament orientation since contraction of antiparallel pairs of actin filaments requires buckling. We tested whether buckling is still necessary for bundle formation in networks with a much smaller number of antiparallel pairs by increasing the bending stiffness of actin filaments 100-fold as before (*κ*_b,A_ = 100×$\kappa_{b,A}^{*}$). We found that networks with barbed ends directed toward +x/+y or +y were still transformed to bundles because contraction in the y-direction does not need to occur in such configurations (Fig 5A and 5C). Filaments in the network with barbed ends directed toward +x/+y initially form only parallel pairs of actin filaments, so they can be aligned in the y-direction (S9A Fig). Filaments forming antiparallel pairs in the x-direction in the network with barbed ends directed toward +y can be aligned in the y-direction via polarity sorting due to the absence of a periodic boundary condition in the x-direction (S9C Fig). Some of the filaments changed their orientation during bundle formation, resulting in antiparallel pairs in the y-direction that were also connected to other actin filaments in a bundle (Fig 5E). Due to suppression of buckling, these pairs cannot contract, so the bundles remained curved rather than straight. Accordingly, forces generated on bundles remained close to zero and even became compressive (i.e. negative) (Fig 5D). By contrast, a network with barbed ends directed toward +x/±y could not form a bundle since the antiparallel pairs of filaments that existed from the beginning were not able to contract (Fig 5B and S9B Fig). Tension generated in these networks was similar to that in networks with isotropic orientations (Fig 5D). Therefore, buckling is not always necessary for the transformation of a network to a bundle. If orientation of actin filaments is highly anisotropic, the transformation can still take place via polarity sorting of filaments by motors. However, tensile forces are not developed on the formed bundles.

### Actin turnover modulates bundle formation and tension generation

**Fig 6:**
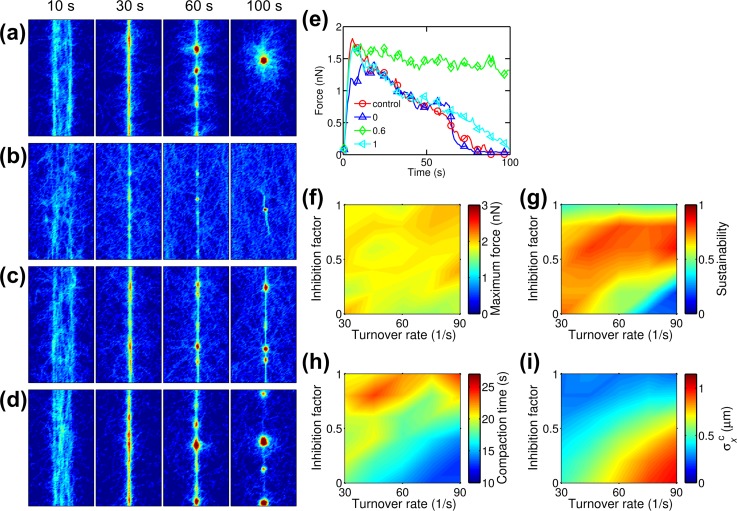
Actin turnover modulates bundle formation and tension generation. Densities of motors (*R*_M_) and ACPs (*R*_ACP_) used in cases shown here are 0.08 and 0.01, respectively. (a-d) Snapshots showing actin density in networks (a) without actin turnover and (b-d) with actin turnover rate (*k*_t,A_) of 60 s^-1^. In networks with actin turnover, depolymerization of actin filaments was inhibited by bound ACPs or motors to an extent determined by inhibition factor (*ξ*_d,A_). *ξ*_d,A_ ranges between 0 (no inhibition of depolymerization) and 1 (complete inhibition). In these examples, *ξ*_d,A_ is (b) 0, (c) 0.6, or (d) 1. (e) Time evolution of tensile forces generated by bundles for cases shown in (a-d). (f) The maximum and (g) sustainability of tension, depending on *k*_t,A_ and *ξ*_d,A_. Maximum tension shows no correlation with *k*_t,A_ and *ξ*_d,A_, whereas sustainability is higher at intermediate range of *ξ*_d,A_. (h) Compaction time. (i) Standard deviation of x positions of actins at compaction time ($\sigma_{x}^{\text{c}}$). With more turnover (i.e. high *k*_t,A_ and low *ξ*_d,A_), bundles form faster, but the formed bundles are more loose.

In our previous study, we demonstrated that actin turnover modulates the buildup and sustainability of tension generated by actomyosin networks [13]. We tested effects of actin turnover on bundle formation and tension generation by imposing actin treadmilling at various rates (*k*_t,A_) under a condition where bundles generate unsustainable tension and eventually form aggregates in the absence of any turnover (*R*_M_ = 0.08 and *R*_ACP_ = 0.01). We additionally assumed that depolymerization of actin filaments can be inhibited by bound ACPs or motors to a different extent [2]. We defined the inhibition factor (*ξ*_d,A_) to represent this effect; with *ξ*_d,A_ = 0, depolymerization is not inhibited at all, whereas inhibition is complete with *ξ*_d,A_ = 1. In a control case without turnover (*k*_t,A_ = 0) and a case with *k*_t,A_ = 60 s^-1^ and *ξ*_d,A_ = 1, bundles became aggregates within 100 s (Fig 6A and 6D), and generated tension fell to nearly zero (Fig 6E). With *k*_t,A_ = 60 s^-1^ and *ξ*_d,A_ = 0, some of the actin filaments in the network formed a thin bundle that was converted into aggregates over time (Fig 6B), and tension ultimately relaxed to zero (Fig 6E). By contrast, with *k*_t,A_ = 60 s^-1^ and *ξ*_d,A_ = 0.6, the bundle was maintained much longer, showing highly sustainable tension (Fig 6C and 6E). We systematically probed the effects of *k*_t,A_ and *ξ*_d,A_ on the maximum and sustainability of tension (Fig 6F and 6G). While maximum tension showed no correlation with *k*_t,A_ and *ξ*_d,A_, sustainability tended to be higher at intermediate levels of *ξ*_d,A_ because too large *ξ*_d,A_ completely inhibits actin turnover, whereas too small *ξ*_d,A_ precludes bundle formation and destabilizes the bundle by ACP unbinding induced by actin turnover. The region with higher sustainability is wider with lower *k*_t,A_, since less turnover occurs at lower *k*_t,A_ at the same level of *ξ*_d,A_. Networks compacted faster with more turnover (i.e. higher *k*_t,A_ and lower *ξ*_d,A_), but formed bundles were loose (Fig 6H and 6I). This agrees with the observation that compaction occurred faster, and more loose bundles formed at lower *R*_ACP_ (Fig 2G and 2H), because more frequent turnover facilitates unbinding of ACPs, leading to a decrease in the number of active ACPs bound on two actin filaments at dynamic equilibrium. Also, with low *ξ*_d,A_, *σ*_*x*_ increased after reaching its minimum (S10 Fig), which corresponds to disintegration of a bundle into a network. However, the increase in *σ*_*x*_ significantly slowed down after some time in several cases, which implies a steady state with coexistence of bundle and network structures as shown in Fig 6C.

At high *R*_ACP_ shown in S11 Fig (*R*_M_ = 0.08 and *R*_ACP_ = 0.1), bundle formation and the maximum tension were both enhanced with slower actin turnover (i.e. lower *k*_t,A_ and higher *ξ*_d,A_). Compaction time, $\sigma_{x}^{\text{c}}$, and *σ*_*x*_ showed similar trends with those in Fig 6 and S10 Fig (S11 and S12 Figs). In this case, the bundle and generated tension are already stable without turnover owing to numerous ACPs. Actin turnover decreases the number of actin filaments involved with bundle formation as can be seen in a change in the diameter of bundles (S11B, S11C and S11D Fig). Thus, the connectivity of filaments in the bundle is deteriorated, resulting in less sustainable tension. In addition, since turnover induces unbinding of ACPs which leads to instability, more motors failed to reach their stall force, leading to smaller maximum tension (S11E Fig). Indeed, $f_{M}^{\max}$ was lower with increasing turnover (S11F Fig). $f_{\mathrm{ACP}}^{\max}$ also decreased with increasing turnover, owing to lower tension and facilitated ACP unbinding by actin turnover. Note that the case with *ξ*_d,A_ = 1 showed more sustained tension than the case without actin turnover. With *ξ*_d,A_ = 1, depolymerization occurs in regions of an actin filament which are not bound to ACPs or motors, thus unnecessary for tension generation. Depolymerized actin can be polymerized at barbed ends of actin filaments, helping sustain tension by increasing a walking distance of motors toward a barbed end. In sum, with an insufficient number of ACPs, actin turnover with intermediate values of *ξ*_d,A_ enhances the stability of bundles and generated tension, whereas with more ACPs, actin turnover plays only a negative role for the stability of bundles and tension.

## Discussion

Structural reorganization of a cross-linked actin network into a bundle occurs in several cellular phenomena, such as formation of transverse arcs at the interface between lamellipodia and lamella. Recent experiments have shown that in the absence of stress fibers, cells can still exert large tensions on surrounding environments due to contractile lamella that contain transverse arcs, implying the significance of transverse arcs in cells as a force generator [23]. To illuminate mechanisms of formation and force generation of transverse arcs, we here presented a computational study regarding transformation of actomyosin networks into bundles under diverse conditions.

Results from this study demonstrate that formation of contractile bundles and force generation in the bundles are tightly regulated by the interplay between concentrations of cytoskeletal elements and the deformability, dynamics, and initial orientation of actin filaments that have not been tested systematically in previous studies. This study is significantly different from our previous study that employed actomyosin bundles preassembled by stacking straight actin filaments in parallel [16] since actin filaments are not stacked merely without any deformation during the morphological transformation. We found that during the transition from a network into a bundle, actin filaments undergo buckling and reorientation in various ways, and a large portion of tension is built during the structural reorganization rather than after bundle formation. In addition, we incorporated systematic variations of initial filament orientation that have not been included in our previous studies [13, 16, 24–27], motivated by observation that transverse arcs located at the interface between lamellipodia and lamella are formed by compaction and realignment of actin filaments with biased orientations within the lamellipodia [28].

We investigated how the density of ACPs and motors and the buckling of actin filaments govern the bundle formation and tension generation. It was found that maximum bundle tension is proportional to motor and ACP densities, whereas sustainability of tension is proportional to ACP density but inversely proportional to motor density. A key factor for determining tension sustainability is how much force is exerted on each ACP because large force can make ACPs unstable by increasing their force-dependent unbinding rate. This is consistent with our previous studies where forces are generated by cortex-like actomyosin networks [19] and preformed bundles [16].

We observed that time required for bundle formation is inversely proportional to motor density but proportional to ACP density. Previous experimental studies showed that condensation of networks into transverse arcs occurs within 20 s [29], which is comparable with the compaction time measured in this study. We also observed that buckling of actin filaments plays an important role in bundle formation, and most of the tension is generated during a transition from a network to a bundle. This is different from our previous study where we found the importance of filament buckling and force generation during contraction of the preformed bundles [16]. In addition, using networks consisting of filaments with biased orientations, we found that buckling should take place in antiparallel pairs of actin filaments initially aligned in the y-direction in order to induce transformation of networks into bundles. If there is not such an antiparallel pair in the y-direction, the transformation is possible without filament buckling. However, development of large tension on a formed bundle is possible only when filament buckling is allowed. In addition, we showed that networks with isotropic filament orientations result in the best bundle formation and the largest tension. Interestingly, even if orientations of actin filaments are too biased to initially have antiparallel pairs of actin filaments, some of the actin filaments change their orientations during network contraction, resulting in antiparallel pairs and formation of bundles. However, compared to the network with isotropic orientations of actin filaments, bundles are loosely formed, and tension is smaller. Since the smaller tension leads to lower force on each ACP, tension is sustained for a longer time.

Also, we probed influences of actin turnover via treadmilling on bundle formation and tension generation as in our previous study. However, we made a new assumption that actin depolymerization rate can be varied by cross-linking points based on previous experimental observations [30]. We observed that actin turnover with moderate inhibition of actin depolymerization by motors and ACPs increases the sustainability of tension and confers structural stability to the bundles at low ACP density. If there is a selective inhibition of depolymerization, the region of a filament that contributes least to the connectivity of bundles (from a pointed end to the first cross-linking point) is depolymerized faster. Depolymerized actin can be polymerized at a barbed end of the same filament or other actin filaments. Since motors walk toward barbed ends, the newly polymerized actin can enable motors to walk further. By contrast, at high ACP density, actin turnover decreases tension sustainability and the stability of formed bundles because the connectivity of the bundles is already maximized by numerous ACPs. Loss of connectivity caused by actin turnover seems more critical than gain of stability from the turnover.

Results from this study support observations from previous studies regarding actomyosin bundles and rings. A recent study showed the importance of architecture and connectivity for the contractility of actomyosin rings [17]. This study showed that each of polarity sorting, sarcomeric contractility, and filament buckling plays an important role at low, intermediate, and high connectivity, respectively. Significant ring contraction was observed only at regimes where sarcomeric contractility or filament buckling becomes important. Too high connectivity or too rigid filaments caused inhibition of filament buckling and ring contraction. Although we did not explore effects of very low connectivity in this study (*R*_ACP_ ≥ 0.01), we observed that buckling takes place less frequently at higher ACP density (Fig 3A), and that suppression of buckling via an increase in filament bending stiffness results in inhibition of contraction (Fig 3B and 3C). All of these are consistent with [17] and other studies showing significance of filament buckling for contraction [31, 32]. Our study also predicted that compaction of an actomyosin network into a bundle is more significant with higher ACP and motor densities. This is in agreement with a recent computational study showing that an actomyosin network exhibits greater contraction and filament alignment with higher densities of motors and ACPs [11]. In addition, another recent computational study found that contraction of random actomyosin arrays mimicking cytokinetic rings is slower with more cross-linkers [33], which is also consistent with our study (Fig 2G).

To summarize, in this study, we systematically investigated how the transformation of the thin actomyosin networks to bundles is regulated by various biophysical factors. We recently demonstrated impacts of severing of actin filaments induced by buckling on rheological behaviors of passive cross-linked actin networks [34]. In future studies, we will include buckling-induced filament severing to test its effects on bundle formation and tension generation.

## Methods

### Brownian dynamics via the langevin equation

Displacements of the segments constituting actin filaments, motors, and ACPs are governed by the Langevin equation with inertia neglected:

$$F_{i}-\zeta_{i}\frac{\text{d}r_{i}}{\text{d}t}+F_{i}^{\text{T}}=0$$ (1)

where **r**_*i*_ is a position vector of the *i*th element, *ζ*_i_ is a drag coefficient, *t* is time, **F**_*i*_ is a deterministic force, and $F_{i}^{\text{T}}$ is a stochastic force satisfying the fluctuation-dissipation theorem [14]:

$$\left\langle F_{i}^{T}\left(t\right)F_{j}^{T}\left(t\right)\right\rangle =\frac{2k_{B}T\zeta_{i}\delta_{ij}}{\Delta t}\delta$$ (2)

where *δ*_*ij*_ is the Kronecker delta, **δ** is a second-order tensor, and Δ*t* = 1.5×10^−5^ s is a time step. Drag coefficients are computed using an approximated form [35]:

$$\zeta_{i}=3\pi \mu r_{\text{c},i}\frac{3+2r_{0,i}/r_{\text{c},i}}{5}$$ (3)

where *μ* is the viscosity of medium, and *r*_0,*i*_ and *r*_c,*i*_ are length and diameter of a segment, respectively. Positions of the various elements are updated using the Euler integration scheme:

$$r_{i}(t+\Delta t)=r_{i}(t)+\frac{\text{d}r_{i}}{\text{d}t}\Delta t=r_{i}(t)+\frac{1}{\zeta_{i}}\left(F_{i}+F_{i}^{\text{T}}\right)\Delta t$$ (4)

### Deterministic forces

Deterministic forces include extensional forces maintaining equilibrium lengths, bending forces maintaining equilibrium angles, and repulsive force between actin segments. Extension and bending of actin, ACP, and motor are governed by harmonic potentials:

$$U_{\text{s}}=\frac{1}{2}\kappa_{\text{s}}{\left(r-r_{0}\right)}^{2}$$ (5)

$$U_{\text{b}}=\frac{1}{2}\kappa_{\text{b}}{\left(\theta -\theta_{0}\right)}^{2}$$ (6)

where *κ*_s_ and *κ*_b_ are extensional and bending stiffness, respectively, *r* is the length of a segment, *θ* is an angle formed by adjacent segments, and the subscript 0 indicates an equilibrium value. An equilibrium length of actin segments (*r*_0,A_ = 140 nm) and an equilibrium angle formed by two adjacent actin segments (*θ*_0,A_ = 0 rad) are maintained by extensional (*κ*_s,A_) and bending stiffness of actin (*κ*_b,A_), respectively. The reference value of *κ*_b,A_ results in persistence length of 9 μm [36]. An equilibrium length of ACP arms (*r*_0,ACP_ = 23.5 nm) and an equilibrium angle between two arms of each ACP (*θ*_0,ACP_ = 0 rad) are maintained by extensional (*κ*_s,ACP_) and bending stiffness of ACPs (*κ*_b,ACP_), respectively. It is assumed that the values of extensional stiffness of a motor backbone (*κ*_s,M1_ and *κ*_s,M2_) keeping an equilibrium length (*r*_s,M1_ = *r*_s,M2_ = 42 nm) are equal to the value of *κ*_s,A_. The bending stiffness of a motor backbone (*κ*_b,M_) keeping the backbone straight (*θ*_0,M_ = 0 rad) is assumed to be larger than *κ*_b,A_. Extension of each motor arm is regulated by stiffness of transverse (*κ*_s,M3_) and longitudinal springs (*κ*_s,M4_). The transverse spring maintains an equilibrium distance (*r*_0,M3_ = 13.5 nm) between an endpoint of a motor backbone and the actin segment where the arm of the motor binds, whereas the longitudinal spring helps maintaining a right angle between the motor arm and the actin segment (*r*_0,M4_ = 0 nm). Forces exerted on actin segments by bound ACPs and motors are distributed onto the barbed and pointed ends of the actin segments as described in our previous work [16].

A repulsive force accounting for volume-exclusion effects between actin segments is represented by following harmonic potential [26]:

$$U_{\text{r}}=\{\begin{array}{c} \frac{1}{2}\kappa_{\text{r}}{\left(r_{12}-r_{\text{c},\text{A}}\right)}^{2}\;\text{if}\;r_{12}<r_{\text{c},\text{A}}\\ \;0\;\text{if}\;r_{12}\geq r_{\text{c},\text{A}} \end{array}$$ (7)

where *κ*_r_ is strength of repulsive force, and *r*_12_ is the minimum distance between two actin segments.

### Kinetics of ACPs and motors

ACPs can bind to binding sites located every 7 nm on actin segments with no preferential angle for binding. ACPs can also unbind from actin filaments in a force-dependent manner following Bell’s equation [15].

$$k_{\text{u},\text{ACP}}=\{\begin{array}{c} k_{\text{u},\text{ACP}}^{0}\exp\left(\frac{\lambda_{\text{u},\text{ACP}}\left|{\vec{F}}_{\text{s},\text{ACP}}\right|}{k_{\text{B}}T}\right)\;\text{if}\;r\geq r_{0,\mathrm{ACP}}\\ k_{\text{u},\text{ACP}}^{0}\;\text{if}\;r<r_{0,\mathrm{ACP}} \end{array}$$ (8)

where $k_{\text{u},\text{ACP}}^{0}$ is the zero-force unbinding rate, *λ*_u,ACP_ represents a sensitivity to applied force, and *k*_*B*_*T* is thermal energy. ${\vec{F}}_{\text{s},\text{ACP}}$ is a vector representing an extensional force acting on an arm of ACP (${\vec{F}}_{\text{s},\text{ACP}}=-\nabla U_{\text{s},\text{ACP}}$). The references values of $k_{\text{u},\text{ACP}}^{0}$ (= 0.115 s^-1^) and *λ*_u,ACP_ (= 1.04×10^−10^ m) are determined to mimic the unbinding behavior of filamin A [37].

Motor arms can bind to binding sites on actin segments with a rate of 40*N*_h_ s^-1^, where *N*_h_ is the number of myosin heads represented by each arm. Walking (*k*_w,M_) and unbinding rates (*k*_u,M_) of the motor arms are determined by the “parallel cluster model” (PCM) [20, 21] to mimic mechanochemical cycle of non-muscle myosin II. *k*_w,M_ and *k*_u,M_ decrease with higher applied load since motors exhibit catch-bond behavior. It was assumed that *k*_w,M_ and *k*_u,M_ are governed by forces exerted on the longitudinal spring of a motor arm that is regulated by *κ*_s,M4_ (${\vec{F}}_{\text{s},\text{M}4}=-\nabla U_{\text{s},\text{M}4}$). Unloaded walking velocity of motors is set to ~140 nm/s and stall force ($f_{M}^{\text{stall}}$) is set to ~5.7 pN.

### Actin dynamics

In the model, actin experiences nucleation, polymerization, and depolymerization. Nucleation corresponds to de novo appearance of one actin segment. Polymerization and depolymerization are implemented by adding and removing one actin segment on filaments, respectively. We simulated treadmilling of actin filaments by imposing equal polymerization and (reference) depolymerization rate at barbed and pointed ends, respectively. A turnover rate indicates how fast an actin filament turns over, which is equal to either polymerization or depolymerization rate. We chose physiologically relevant turnover rates (30–120 s^-1^). A nucleation rate is also adjusted to maintain a relatively constant actin filament length. We assumed that actin nucleation takes place in the y-direction within a bundle.

It is assumed that depolymerization can be inhibited by bound ACPs or motors [30]; an inhibition factor ranging between 0 and 1 (*ξ*_d,A_) determines the extent of inhibition:

$$k_{\text{d},\text{A}}=k_{\text{d},\text{A}}^{0}\left(1-\xi_{\text{d},\text{A}}\right)$$ (9)

where *k*^0^_d,A_ and *k*_d,A_ are reference and adjusted depolymerization rates at a barbed end or a pointed end. Thus, *ξ*_d,A_ = 0 corresponds to no depolymerization inhibition, whereas *ξ*_d,A_ = 1 means complete inhibition.

### Network assembly

We used a 3D rectangular computational domain (4 × 8 × 0.5 μm) with a periodic boundary condition in the y-direction. Self-assembly of actin filaments, ACPs, and motors in the domain results in a homogenous actomyosin network. During the network assembly, actin monomers are nucleated and polymerized into filaments. When creating anisotropic networks, direction of nucleation is controlled so that actin filaments lie along desired directions after network assembly. Motors are assembled into thick filaments, and motor arms bind to actin filaments without walking motion. ACPs bind to actin filaments to form functional cross-links between pairs of actin filaments. Due to the fixed ratio of nucleation rate to turnover rate, the average length of actin filaments is maintained at ~1.56 μm. After the network assembly, motors start walking on actin filaments, and the nucleation rate is dynamically controlled to maintain the average filament length at a constant level. Actin monomer concentration (*C*_A_) is 40 μM for all cases.

### Measurement of force and sustainability

To measure tension generated by a bundle, we consider 10 cross-sections that are regularly located in the computational domain in the y-direction. Tension is calculated by summing the normal component of extensional forces of all constituents crossing a cross-section. We repeat this calculation on 10 cross-sections and compute the average. Sustainability of the tension is calculated in the same manner as in [13].

Microscopic forces acting on ACPs ($f_{\mathrm{ACP}}^{\max}$) and motors ($f_{M}^{\max}$) are evaluated when tension reaches a maximum:

$$f_{\mathrm{ACP}}^{\max}={\vec{F}}_{\text{s},\text{ACP}}\cdot \vec{u}$$ (10)

$$f_{M}^{\max}={\vec{F}}_{\text{s},\text{M}4}\cdot \vec{u}/N_{h}$$ (11)

where $\vec{u}$ is a unit vector directed toward a barbed end of actin filaments. Note that ${\vec{F}}_{\text{s},\text{ACP}}$ and ${\vec{F}}_{\text{s},\text{M}4}$ are directed from the center of ACP or the endpoint on a motor backbone to a binding point on an actin filament where ACP or motor is currently bound, and that $f_{\mathrm{ACP}}^{\max}$ and $f_{M}^{\max}$ are positive when the force vectors are directed toward barbed ends. Most of $f_{M}^{\max}$ values are positive because motor arms walk toward barbed ends, and because the unbinding rate of the motor arm defined by the PCM model is assumed to be very large when ${\vec{F}}_{\text{s},\text{M}4}$ is directed toward a pointed end. By contrast, values of $f_{\mathrm{ACP}}^{\max}$ show largely symmetric distribution due to the absence of walking motion and unbinding rate independent of the direction of ${\vec{F}}_{\text{s},\text{ACP}}$. However, there is slightly higher population on negative values of $f_{\mathrm{ACP}}^{\max}$ since ACPs sustain forces exerted by motors which are mostly positive.

### Quantification of formation time and shape of bundles

We measured time evolution of standard deviation of x positions of actins (*σ*_*x*_). *σ*_*x*_ decreases as a bundle forms and then either remains relatively constant until the end of the simulations or increases slowly over time if the bundle is disintegrated. As a measure of how fast a network compacts into a bundle, we define the compaction time as time when the rate of change in *σ*_*x*_ becomes larger than 0.01 × (the average rate of change in *σ*_*x*_ for the first 5s). In addition, we used the magnitude of the standard deviation at the same time point ($\sigma_{x}^{\text{c}}$) as a measure of how tightly the bundle is formed in the x-direction.

## Supporting Information

S1 Table List and definition of terms. (DOCX)

S2 Table List of parameters employed in the model. (DOCX)

S1 Fig Time evolution of standard deviation of x positions of actins (*σ*_*x*_) for the cases shown in Fig 2. ACP density used in these cases is (a) 0.01, (b) 0.018, (c) 0.032, (d) 0.056, and (e) 0.1. Motor density is 0.0008 (red), 0.0026 (blue), 0.008 (green), 0.026 (cyan), and 0.08 (black).
(TIF)

S2 Fig Buckling of actin filaments in the case shown in Fig 2B. (a) The ratio of end-to-end distance to contour length of two selected actin filaments. The actin filament represented by red experienced a sequence of buckling events at around *t* = 8, 25, 50, and 80 s, whereas the actin filament represented by blue underwent buckling at around *t* = 8 s and was straightened at around *t* = 75 s. (b, c) Visualization of (b) subsequent buckling events and (c) straightening of buckled actin filaments shown in (a). Solid circles located at the ends of the actin filaments represent their barbed ends.
(TIF)

S3 Fig Effects of duty ratio of motors on bundle formation and tension generation. Densities of motors (*R*_M_) and ACPs (*R*_ACP_) are 0.08 and 0.1, respectively. Compared to the reference case with the same *R*_M_ and *R*_ACP_ (Fig 2B), stall force of motors was decreased from 5.7 pN to 5.3 pN, and unbinding rate was increased from 0.049 s^-1^ to 0.49 s^-1^. (a) Snapshots showing actin density in the networks at *t* = 10, 30, and 60 s. A bundle forms well as in the reference case. (b-c) Time evolutions of (b) tension and (c) standard deviation of x positions of actins (*σ*_*x*_) show similar tendency to that in the reference case.
(TIF)

S4 Fig Time evolution of standard deviation of x positions of actins (*σ*_*x*_) for the cases shown in Fig 3. Density of ACPs used in these cases is (a) 0.01, (b) 0.032, and (c) 0.1. Motor density is 0.0008 (red), 0.0026 (blue), 0.008 (green), 0.026 (cyan), and 0.08 (black).
(TIF)

S5 Fig Rotation of actin filaments in the case shown in Fig 4C. (a) Time evolution of orientation of two selected actin filaments. At around *t* = 20 s, both actin filaments rotate by about 180° (b, c) Visualization of rotation of the actin filaments shown in (a). Solid circles located at the ends of the actin filaments represent their barbed ends.
(TIF)

S6 Fig Effects of initial orientation of diagonally nuclearized actin filaments on bundle formation and tension generation. Densities of motors and ACPs used in cases shown here are 0.08 and 0.01, respectively. (a-d) (1st column) Orientations where barbed ends of actin filaments in networks are initially directed. Red lines on the circles located at the bottom-right corner represent the orientations. Arrows in the boxes represent examples of filaments with corresponding initial orientations. (2nd, 3rd, 4th columns) Snapshots showing actin density in the networks at *t* = 0, 10, and 40 s with initial orientation indicated in the 1st column. (e) Time evolution of tension for cases shown in (a-d). (f) Time evolution of orientations of selected actin filaments in the case shown in (a). (g) Time evolution of a fraction of antiparallel filament pairs for cases shown in (a-d). (h) Number of buckling events occurring during simulations for cases shown in (a-d).
(TIF)

S7 Fig Influences of initial orientation of perpendicularly nuclearized actin filaments on bundle formation and tension generation. Densities of motors and ACPs used in cases shown here are 0.08 and 0.01, respectively. (a-d) (1st column) Orientations where barbed ends of actin filaments in networks are initially directed. (2nd, 3rd, 4th columns) Snapshots showing actin density in the networks at *t* = 0, 10, and 40 s with initial orientation indicated in the 1st column. (e) Time evolution of tension for cases shown in (a-d). (f) Time evolution of orientation of selected actin filaments from case shown in (a). (g) Time evolution of a fraction of antiparallel filament pairs for cases shown in (a-d). (h) Number of buckling events occurring during simulations for cases shown in (a-d).
(TIF)

S8 Fig Effects of initial orientation of actin filaments on bundle formation and tension generation in networks with numerous ACPs. Densities of motors and ACPs used in cases shown here are 0.08 and 0.1, respectively. (a-c) (1st column) Orientations where barbed ends of actin filaments in networks are initially directed. (2nd, 3rd, 4th columns) Snapshots showing actin density in the networks at *t* = 10, 30, and 60 s with initial orientation indicated in the 1st column. (5th column) Initial and final orientations of actin filaments. Final orientation indicates orientation of filaments measured at a time point when compaction time is defined. (d) Time evolution of tension for cases with biased initial orientations shown in (a-c) and isotropic initial orientation. (e) Time evolution of a fraction of antiparallel filament pairs for cases shown in (d).
(TIF)

S9 Fig In networks with biased filament orientations, bundles can form without buckling of actin filaments. Schematic diagrams show actin filaments and motors initially directed toward (a) +x/+y, (b) +x/±y, and (c) ±x/+y as in cases shown in Fig 4A–4C. Teal and red represent actin filaments and motors, respectively, whereas ACPs are not shown for simplicity. Because the periodic boundary condition exists only in the y-direction, actin filaments tend to be aligned in the y-direction. (a) Most of the actin filaments oriented toward +x/+y are aligned in parallel via polarity sorting. (b) Antiparallel pairs of actin filaments initially oriented relatively in the y-direction can be aligned well in the y-direction. However, the alignment results in the buildup of compressive forces on the actin filaments unlike in other cases. If bending stiffness of actin filaments is low enough, the actin filaments are buckled and oriented in the y-direction. If buckling is suppressed due to large bending stiffness, the actin filaments cannot be oriented in the y-direction. (c) Antiparallel pairs of actin filaments initially oriented relatively in the x-direction can be aligned in the y-direction via polarity sorting.
(TIF)

S10 Fig Time evolution of standard deviation of x positions of actins (*σ*_*x*_) in the cases shown in Fig 6. The turnover rate used in these cases is (a) 30 s^-1^, (b) 45 s^-1^, (c) 60 s^-1^, (d) 75 s^-1^, and (e) 90 s^-1^. The inhibition factor is 0 (red), 0.2 (blue), 0.4 (green), 0.6 (cyan), 0.8 (black), and 1 (magenta).
(TIF)

S11 FigImpacts of actin turnover on bundle formation and tension generation in networks with numerous ACPs.Densities of motors and ACPs used in cases shown here are 0.08 and 0.1, respectively. (a-d) Snapshots showing actin density in networks (a) without actin turnover and (b-d) with actin turnover rate (*k*_t,A_) of 60 s^-1^. In networks with actin turnover, depolymerization of actin filaments was inhibited by bound ACPs or motors to an extent determined by inhibition factor (*ξ*_d,A_). *ξ*_d,A_ ranges between 0 (no inhibition of depolymerization) and 1 (complete inhibition). In these examples, *ξ*_d,A_ is (b) 0, (c) 0.5, or (d) 1. (e) Time evolution of tensile forces generated by bundles for cases shown in (a-d). (f) Distribution of forces exerted on motors ($f_{M}^{\max}$) and ACPs ($f_{\mathrm{ACP}}^{\max}$) measured at peak tension for cases shown in (a-d). The gray dashed line indicates stall force of motors (5.7pN). The legend is shared with (e). (g) The maximum and (h) sustainability of tension, depending on *k*_t,A_ and *ξ*_d,A_. (i) Compaction time. (j) Standard deviation of x positions of actins at the compaction time ($\sigma_{x}^{\text{c}}$).(TIF)

S12 Fig Time evolution of standard deviation of x positions of actins (*σ*_*x*_) in the cases shown in S11 Fig. The turnover rate used in these cases is (a) 60 s^-1^, (b) 90 s^-1^, and (c) 120 s^-1^. The inhibition factor is 0 (red), 0.25 (blue), 0.5 (green), 0.75 (cyan), and 1 (black).
(TIF)

## References

[1] Gardel ML, Kasza KE, Brangwynne CP, Liu J, Weitz DA. Mechanical response of cytoskeletal networks. Methods Cell Biol. 2008;89:487–519. 10.1016/S0091-679X(08)00619-5 19118688 PMC4456006

[2] Murrell M, Oakes PW, Lenz M, Gardel ML. Forcing cells into shape: the mechanics of actomyosin contractility. Nat Rev Mol Cell Biol. 2015;16(8):486–498. 10.1038/nrm4012 26130009 PMC7443980

[3] Reichl EM, Ren Y, Morphew MK, Delannoy M, Effler JC, Girard KD, et al Interactions between myosin and actin crosslinkers control cytokinesis contractility dynamics and mechanics. Curr Biol. 2008;18(7):471–80. 10.1016/j.cub.2008.02.056 18372178 PMC2361134

[4] Bidone TC, Tang H, Vavylonis D. Dynamic network morphology and tension buildup in a 3D model of cytokinetic ring assembly. Biophys J. 2014;107(11):2618–28. 10.1016/j.bpj.2014.10.034 25468341 PMC4255221

[5] Laporte D, Ojkic N, Vavylonis D, Wu JQ. alpha-Actinin and fimbrin cooperate with myosin II to organize actomyosin bundles during contractile-ring assembly. Mol Biol Cell. 2012;23(16):3094–110. 10.1091/mbc.E12-02-0123 22740629 PMC3418305

[6] Tojkander S, Gateva G, Lappalainen P. Actin stress fibers–assembly, dynamics and biological roles. J Cell Sci. 2012;125(8):1855–1864. 22544950 10.1242/jcs.098087

[7] Burnette DT, Manley S, Sengupta P, Sougrat R, Davidson MW, Kachar B, et al A role for actin arcs in the leading-edge advance of migrating cells. Nat Cell Biol. 2011;13(4):371–382. 10.1038/ncb2205 21423177 PMC3646481

[8] Hotulainen P, Lappalainen P. Stress fibers are generated by two distinct actin assembly mechanisms in motile cells. J Cell Biol. 2006;173(3):383–94. 10.1083/jcb.200511093 16651381 PMC2063839

[9] Gordon D, Bernheim-Groswasser A, Keasar C, Farago O. Hierarchical self-organization of cytoskeletal active networks. Phys Biol. 2012;9(2):026005 10.1088/1478-3975/9/2/026005 22476003

[10] Inoue Y, Tsuda S, Nakagawa K, Hojo M, Adachi T. Modeling myosin-dependent rearrangement and force generation in an actomyosin network. J Theor Biol. 2011;281(1):65–73. 10.1016/j.jtbi.2011.04.004 21514305

[11] Popov K, Komianos J, Papoian GA. MEDYAN: Mechanochemical Simulations of Contraction and Polarity Alignment in Actomyosin Networks. PLoS Comput Biol. 2016;12(4):e1004877 10.1371/journal.pcbi.1004877 27120189 PMC4847874

[12] Lenz M. Geometrical origins of contractility in disordered actomyosin networks. Phys Rev X. 2014;4(4):041002.

[13] Mak M, Zaman MH, Kamm RD, Kim T. Interplay of active processes modulates tension and drives phase transition in self-renewing, motor-driven cytoskeletal networks. Nat Commun. 2016;7:10323 10.1038/ncomms10323 26744226 PMC4714927

[14] Underhill PT, Doyle PS. On the coarse-graining of polymers into bead-spring chains. J Nonnewton Fluid Mech. 2004;122(1):3–31.

[15] Bell GI. Models for the specific adhesion of cells to cells. Science. 1978;200(4342):618–27. 347575 10.1126/science.347575

[16] Kim T. Determinants of contractile forces generated in disorganized actomyosin bundles. Biomech Model Mechanobiol. 2015;14(2):345–55. 10.1007/s10237-014-0608-2 25103419

[17] Ennomani H, Letort G, Guérin C, Martiel J-L, Cao W, Nédélec F, et al Architecture and Connectivity Govern Actin Network Contractility. Curr Biol. 2016;26(5):616–26. 10.1016/j.cub.2015.12.069 26898468 PMC4959279

[18] Reymann A-C, Boujemaa-Paterski R, Martiel J-L, Guérin C, Cao W, Chin HF, et al Actin network architecture can determine myosin motor activity. Science. 2012;336(6086):1310–4. 10.1126/science.1221708 22679097 PMC3649007

[19] Jung W, Murrell MP, Kim T. F-actin cross-linking enhances the stability of force generation in disordered actomyosin networks. Comput Part Mech. 2015;2(4):317–27.

[20] Erdmann T, Schwarz US. Stochastic force generation by small ensembles of myosin II motors. Phys Rev Lett. 2012;108(18):188101 10.1103/PhysRevLett.108.188101 22681120

[21] Erdmann T, Albert PJ, Schwarz US. Stochastic dynamics of small ensembles of non-processive molecular motors: The parallel cluster model. J Chem Phys. 2013;139(17):175104 10.1063/1.4827497 24206337

[22] Cramer LP, Siebert M, Mitchison TJ. Identification of novel graded polarity actin filament bundles in locomoting heart fibroblasts: implications for the generation of motile force. J Cell Biol. 1997;136(6):1287–305. 9087444 10.1083/jcb.136.6.1287 PMC2132518

[23] Oakes PW, Beckham Y, Stricker J, Gardel ML. Tension is required but not sufficient for focal adhesion maturation without a stress fiber template. J Cell Biol. 2012;196(3):363–74. 10.1083/jcb.201107042 22291038 PMC3275371

[24] Borau C, Kim T, Bidone T, García-Aznar JM, Kamm RD. Dynamic mechanisms of cell rigidity sensing: insights from a computational model of actomyosin networks. PLoS One. 2012;7(11):e49174 10.1371/journal.pone.0049174 23139838 PMC3489786

[25] Kim T, Hwang W, Kamm RD. Dynamic role of cross-linking proteins in actin rheology. Biophys J. 2011;101(7):1597–603. 10.1016/j.bpj.2011.08.033 21961585 PMC3183755

[26] Kim T, Hwang W, Lee H, Kamm RD. Computational analysis of viscoelastic properties of crosslinked actin networks. PLoS Comput Biol. 2009;5(7):e1000439 10.1371/journal.pcbi.1000439 19609348 PMC2703781

[27] Bidone TC, Kim T, Deriu MA, Morbiducci U, Kamm RD. Multiscale impact of nucleotides and cations on the conformational equilibrium, elasticity and rheology of actin filaments and crosslinked networks. Biomech Model Mechanobiol. 2015;14(5):1143–55. 10.1007/s10237-015-0660-6 25708806

[28] Verkhovsky AB, Chaga OY, Schaub S, Svitkina TM, Meister J-J, Borisy GG. Orientational order of the lamellipodial actin network as demonstrated in living motile cells. Mol Biol Cell. 2003;14(11):4667–75. 10.1091/mbc.E02-10-0630 13679520 PMC266781

[29] Heath JP. Behaviour and structure of the leading lamella in moving fibroblasts. I. Occurrence and centripetal movement of arc-shaped microfilament bundles beneath the dorsal cell surface. J Cell Sci. 1983;60:331–54. 6348051 10.1242/jcs.60.1.331

[30] Biron D, Moses E. The effect of α-actinin on the length distribution of f-actin. Biophys J. 2004;86(5):3284–90. 10.1016/S0006-3495(04)74376-3 15111441 PMC1304193

[31] Lenz M, Gardel ML, Dinner AR. Requirements for contractility in disordered cytoskeletal bundles. New J Phys. 2012;14(3):033037. 10.1088/1367-2630/14/3/033037 PMC3496381 23155355

[32] Lenz M, Thoresen T, Gardel ML, Dinner AR. Contractile units in disordered actomyosin bundles arise from F-actin buckling. Phys Rev Lett. 2012;108(23):238107 10.1103/PhysRevLett.108.238107 23003998 PMC4447086

[33] Oelz DB, Rubinstein BY, Mogilner A. A Combination of Actin Treadmilling and Cross-Linking Drives Contraction of Random Actomyosin Arrays. Biophys J. 2015;109(9):1818–1829. 10.1016/j.bpj.2015.09.013 26536259 PMC4643270

[34] Jung W, Murrell MP, Kim T. F-Actin Fragmentation Induces Distinct Mechanisms of Stress Relaxation in the Actin Cytoskeleton. ACS Macro Lett. 2016;5:641–5. 10.1021/acsmacrolett.6b00232 35614663

[35] Clift R, Grace JR, Weber ME. Bubbles, drops, and particles: Courier Corporation; 2005.

[36] Isambert H, Venier P, Maggs AC, Fattoum A, Kassab R, Pantaloni D, et al Flexibility of actin filaments derived from thermal fluctuations. Effect of bound nucleotide, phalloidin, and muscle regulatory proteins. J Biol Chem. 1995;270(19):11437–44. 7744781 10.1074/jbc.270.19.11437

[37] Ferrer JM, Lee H, Chen J, Pelz B, Nakamura F, Kamm RD, et al Measuring molecular rupture forces between single actin filaments and actin-binding proteins. Proc Natl Acad Sci U S A. 2008;105(27):9221–6. 10.1073/pnas.0706124105 18591676 PMC2453742

