# Supplementary material for: Morphological Transformation and Force Generation of Active Cytoskeletal Networks
Source: PLoS Comput Biol. 2017 Jan 23;13(1):e1005277. doi: 10.1371/journal.pcbi.1005277 (PMC5256887; doi:10.1371/journal.pcbi.1005277)
Supplement: S1 Table — (DOCX) [file pcbi.1005277.s001.docx]

| **Term** | **Definition** |
| --- | --- |
| ACP | Actin cross-linking protein that transiently connects a pair of actin filaments |
| Sustainability | A measure of how well tension generated by a network is maintained over time. Sustainability is 1 if tension does not decrease at all after reaching the peak value, whereas it is 0 for the case where tension drops most rapidly between all simulated cases in each figure. |
| $f_{\text{M}}^{\max}$ | Force exerted on each motor when tension reaches its peak |
| $f_{\text{ACP}}^{\max}$ | Force exerted on each ACP when tension reaches its peak |
| *σ_x_* | Standard deviation of x positions of actins |
| Compaction time | A measure of how fast a network compacts into a bundle. It is defined as time when the rate of change in *σ_x_* becomes larger than 0.01 × (the average rate of change in *σ_x_* during first 5s). |
| $\sigma_{x}^{\text{c}}$ | Standard deviation of x positions of actins at compaction time |
| Inhibition factor  (*ξ*_d,A_) | Extent to which actin depolymerization is inhibited by bound ACPs and motors. When depolymerization of an actin segment with any bound ACP or motor is considered, “1- *ξ*_d,A_” is multiplied to a reference depolymerization rate for partial or complete inhibition. |
