# Supplementary material for: Morphological Transformation and Force Generation of Active Cytoskeletal Networks
Source: PLoS Comput Biol. 2017 Jan 23;13(1):e1005277. doi: 10.1371/journal.pcbi.1005277 (PMC5256887; doi:10.1371/journal.pcbi.1005277)
Supplement: S2 Table — (DOCX) [file pcbi.1005277.s002.docx]

| **Symbol** | **Definition** | **Value** |
| --- | --- | --- |
| *r*_0,A_ | Length of an actin segment | 1.4×10^-7^ [m] |
| *r*_c,A_ | Diameter of an actin segment | 7.0×10^-9^ [m] [3] |
| *θ*_0,A_ | Bending angle formed by adjacent actin segments | 0 [rad] |
| *κ*_s,A_ | Extensional stiffness of actin filament | 1.69×10^-2^ [N/m] |
| *κ*_b,A_ | Bending stiffness of actin filament | 2.64×10^-19^ [N·m] [1] |
| *r*_0,ACP_ | Length of an ACP arm | 2.0×10^-8^ [m] [4] |
| *r*_c,ACP_ | Diameter of an ACP arm | 1.0×10^-8^ [m] |
| *θ*_0,ACP_ | Bending angle formed by two ACP arms | 0 [rad] |
| *κ*_s,ACP_ | Extensional stiffness of ACP | 2.0×10^-3^ [N/m] |
| *κ*_b,ACP_ | Bending stiffness of ACP | 0 [N·m] |
| *r*_0,M1_ | Length of a bare zone of motor backbone | 4.2×10^-8^ [m] |
| *r*_0,M2_ | Length of a side segment of motor backbone | 4.2×10^-8^ [m] |
| *θ*_0,M_ | Bending angle formed by segments of motor backbone | 0 [rad] |
| *κ*_s,M1_ | Extensional stiffness of a bare zone | 1.69×10^-2^ [N/m] |
| *κ*_s,M2_ | Extensional stiffness of a side segment | 1.69×10^-2^ [N/m] |
| *κ*_b,M_ | Bending stiffness of motor backbone | 5.07×10^-18^ [N·m] |
| *r*_0,M3_ | Length of a motor arm | 1.0×10^-8^ [m] |
| *r*_c,M_ | Diameter of a motor arm | 1.0×10^-8^ [m] |
| *κ*_s,M3_ | Extensional stiffness 1 of a motor arm | 1.0×10^-3^ [N/m] |
| *κ*_s,M4_ | Extensional stiffness 2 of a motor arm | 1.0×10^-3^ [N/m] |
| *N*_h_ | Number of heads represented by a single motor arm | 8 |
| *N*_a_ | Number of arms per motor | 8 |
| *κ*_r_ | Strength of repulsive force | 1.69×10^-3^ [N/m] |
| *C*_A_ | Actin concentration | 40 [μM] |
| *R*_M_ | Ratio of motor concentration to *C*_A_ | 0.0008-0.08 |
| *R*_ACP_ | Ratio of ACP concentration to *C*_A_ | 0.01-0.1 |
| <*L*_f_> | Average length of actin filaments | 1.56 [μm] |
| Δ*t* | Time step | 1.5×10^-5^ [s] |
| *μ* | Viscosity of medium | 8.6×10^-1^ [kg/m·s] |
| *k*_B_*T* | Thermal energy | 4.142×10^-21^ [J] |
| *k*_b,ACP_ | Binding rate of ACP | 1000 [1/μM·s] |
| $k_{\text{u,ACP}}^{0}$ | Zero-force unbinding rate coefficient of ACP | 0.115 [s^-1^] (=$k_{\text{u,ACP}}^{0*}$) [2] |
| *λ*_u,ACP_ | Force sensitivity of ACP unbinding | 1.04×10^-10^ [m] [2] |
| *k*_t,A_ | Actin turnover rate | 30 - 120 [s^-1^] |
| $k_{\text{d,A}}^{0}$ | Reference actin depolymerization rate | 30 - 120 [s^-1^] |
| *ξ*_d,A_ | Inhibition factor for actin depolymerization | 0 - 1 |

**References**

1. Isambert H, Venier P, Maggs AC, Fattoum A, Kassab R, Pantaloni D, et al. Flexibility of actin filaments derived from thermal fluctuations. Effect of bound nucleotide, phalloidin, and muscle regulatory proteins. J Biol Chem. 1995;270(19):11437-44.

2. Ferrer JM, Lee H, Chen J, Pelz B, Nakamura F, Kamm RD, et al. Measuring molecular rupture forces between single actin filaments and actin-binding proteins. Proc Natl Acad Sci U S A. 2008;105(27):9221-6.

3. Kishino A, Yanagida T. Force measurements by micromanipulation of a single actin filament by glass needles. Nature. 1988;334:74-6.

4. Meyer RK, Aebi U. Bundling of actin filaments by alpha-actinin depends on its molecular length. J Cell Biol. 1990;110(6):2013-24.
