# Supplementary figures and images for: Morphological Transformation and Force Generation of Active Cytoskeletal Networks
Source: PLoS Comput Biol. 2017 Jan 23;13(1):e1005277. doi: 10.1371/journal.pcbi.1005277 (PMC5256887; doi:10.1371/journal.pcbi.1005277)

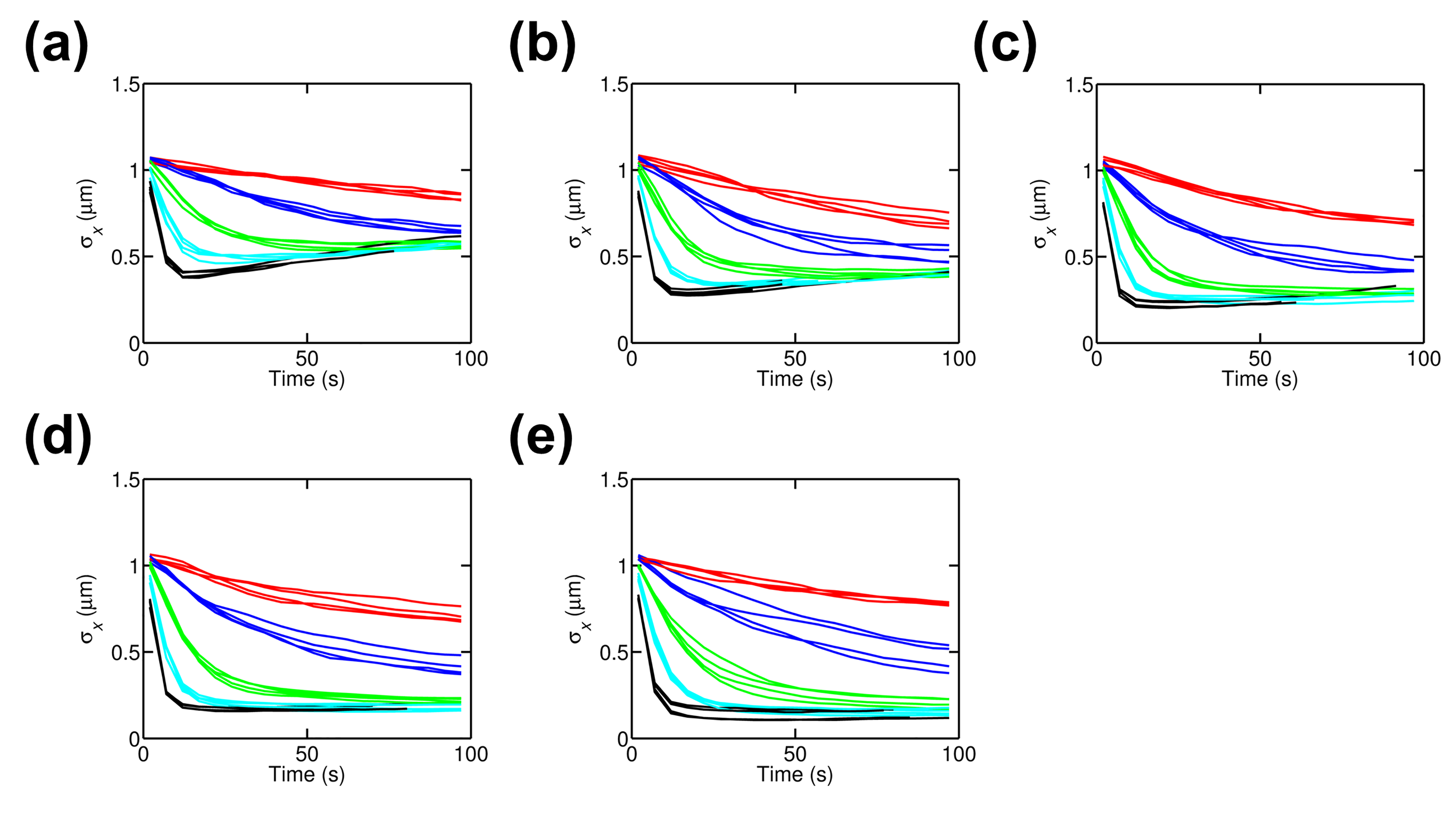

Supplement: S1 Fig — ACP density used in these cases is (a) 0.01, (b) 0.018, (c) 0.032, (d) 0.056, and (e) 0.1. Motor density is 0.0008 (red), 0.0026 (blue), 0.008 (green), 0.026 (cyan), and 0.08 (black). (TIF) [file pcbi.1005277.s003.TIF]

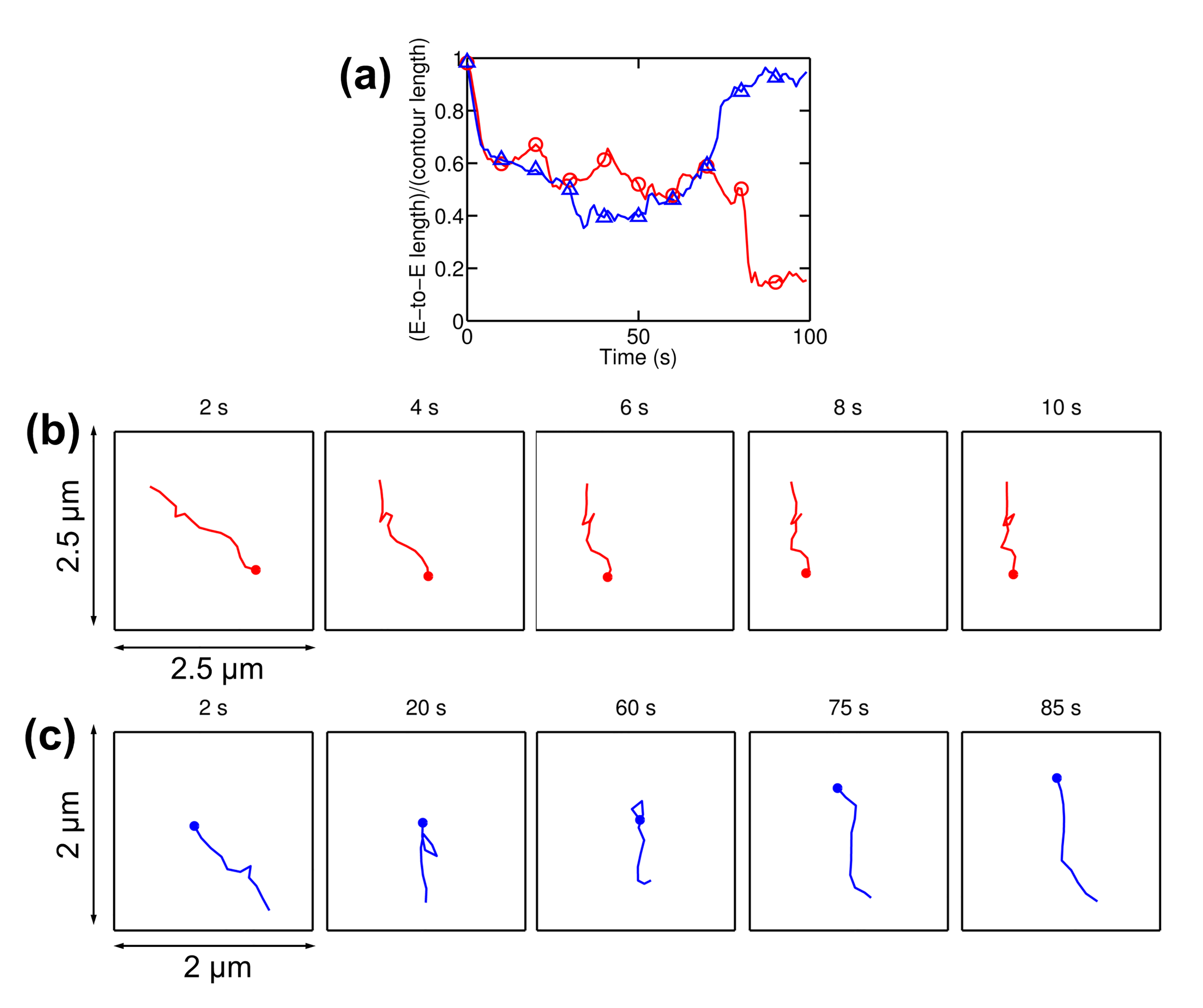

Supplement: S2 Fig — (a) The ratio of end-to-end distance to contour length of two selected actin filaments. The actin filament represented by red experienced a sequence of buckling events at around t = 8, 25, 50, and 80 s, whereas the actin filament represented by blue underwent buckling at around t = 8 s and was straightened at around t = 75 s. (b, c) Visualization of (b) subsequent buckling events and (c) straightening of buckled actin filaments shown in (a). Solid circles located at the ends of the actin filaments represent their barbed ends. (TIF) [file pcbi.1005277.s004.TIF]

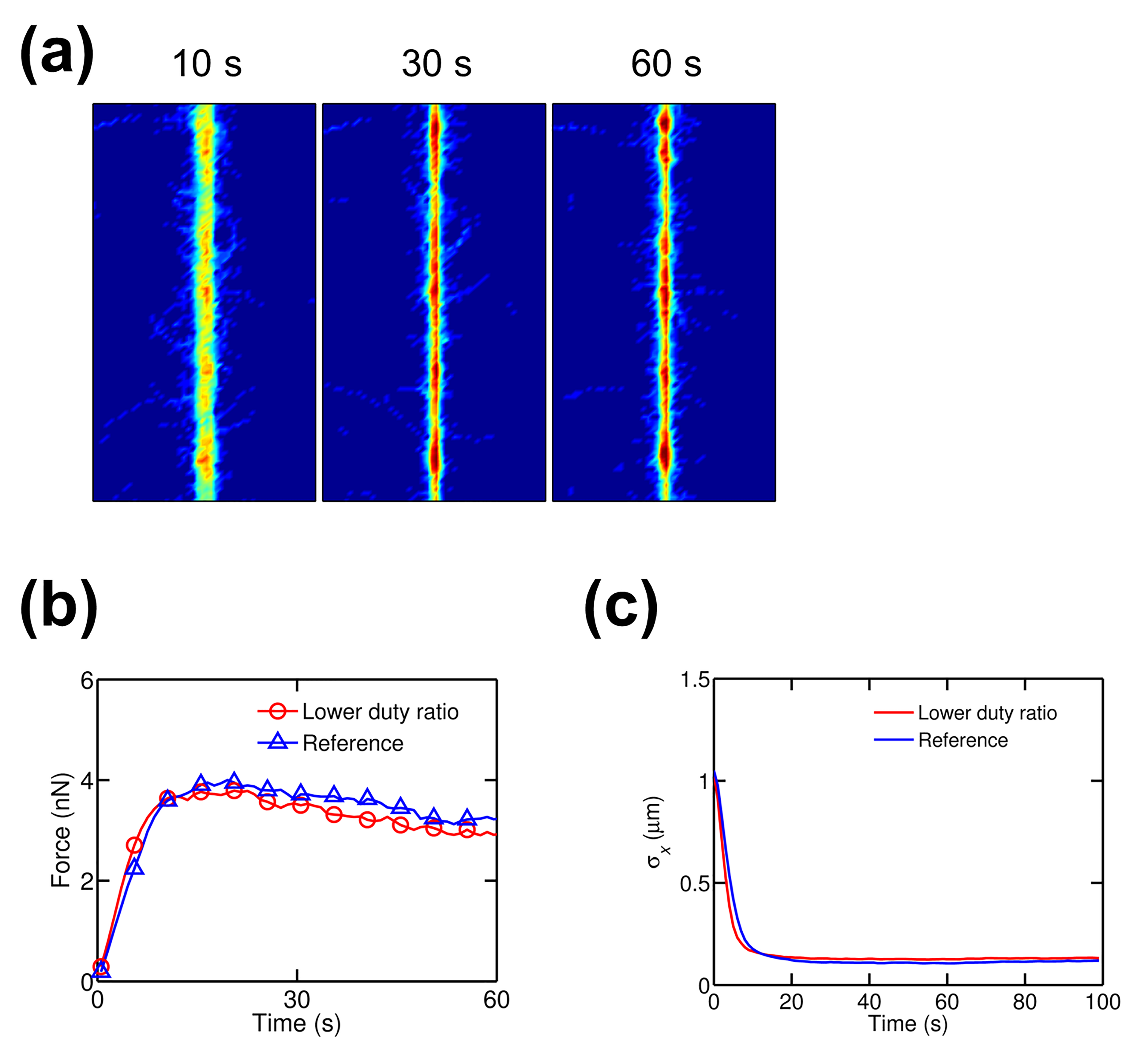

Supplement: S3 Fig — Densities of motors (RM) and ACPs (RACP) are 0.08 and 0.1, respectively. Compared to the reference case with the same RM and RACP (Fig 2B), stall force of motors was decreased from 5.7 pN to 5.3 pN, and unbinding rate was increased from 0.049 s-1 to 0.49 s-1. (a) Snapshots showing actin density in the networks at t = 10, 30, and 60 s. A bundle forms well as in the reference case. (b-c) Time evolutions of (b) tension and (c) standard deviation of x positions of actins (σx) show similar tendency to that in the reference case. (TIF) [file pcbi.1005277.s005.TIF]

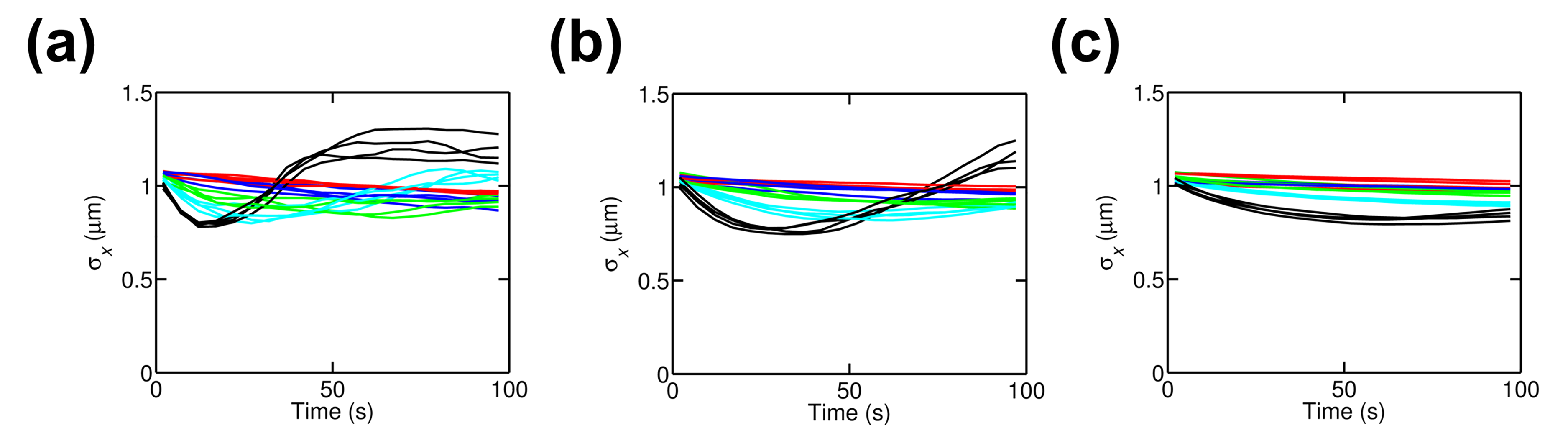

Supplement: S4 Fig — Density of ACPs used in these cases is (a) 0.01, (b) 0.032, and (c) 0.1. Motor density is 0.0008 (red), 0.0026 (blue), 0.008 (green), 0.026 (cyan), and 0.08 (black). (TIF) [file pcbi.1005277.s006.TIF]

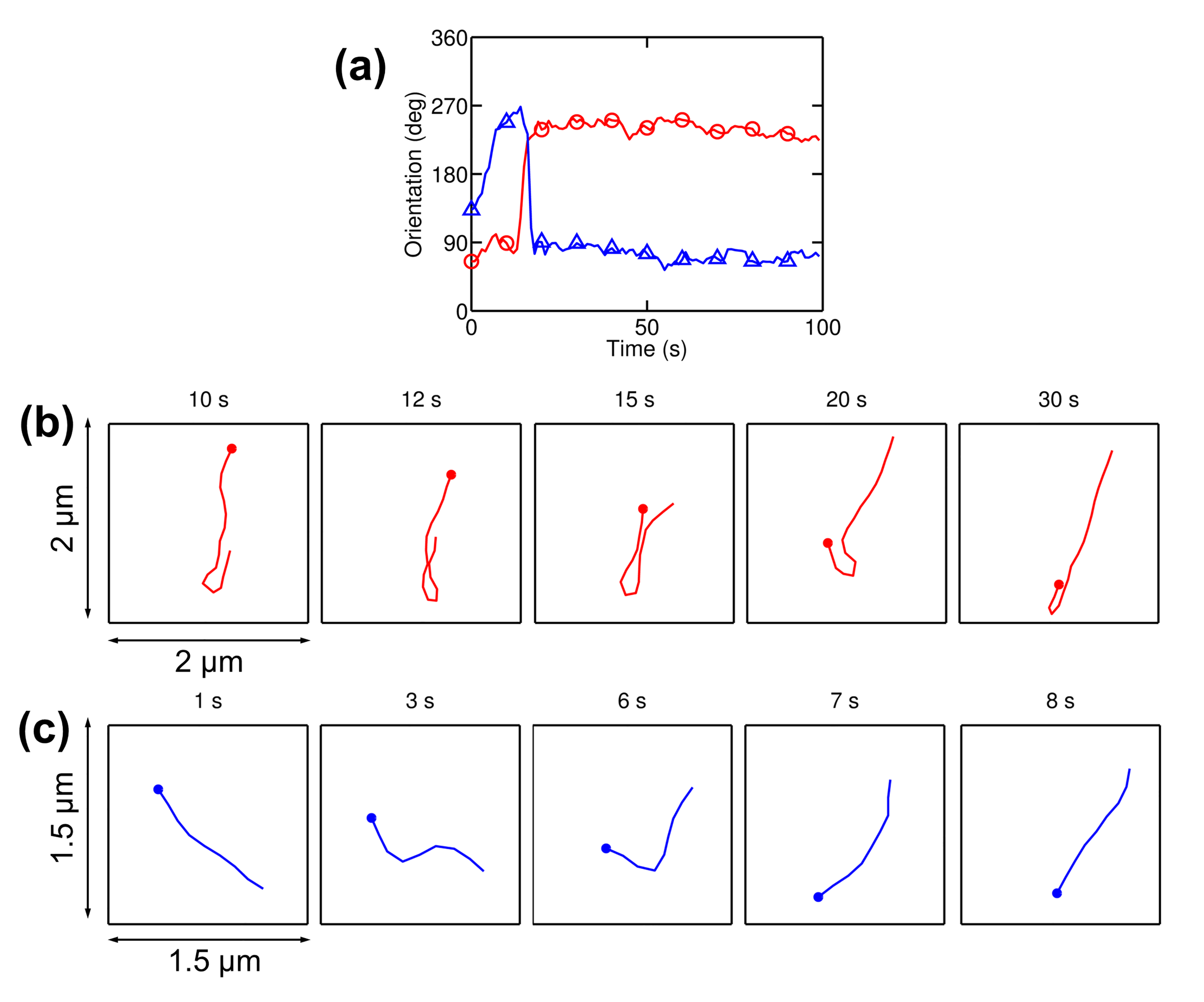

Supplement: S5 Fig — (a) Time evolution of orientation of two selected actin filaments. At around t = 20 s, both actin filaments rotate by about 180° (b, c) Visualization of rotation of the actin filaments shown in (a). Solid circles located at the ends of the actin filaments represent their barbed ends. (TIF) [file pcbi.1005277.s007.TIF]

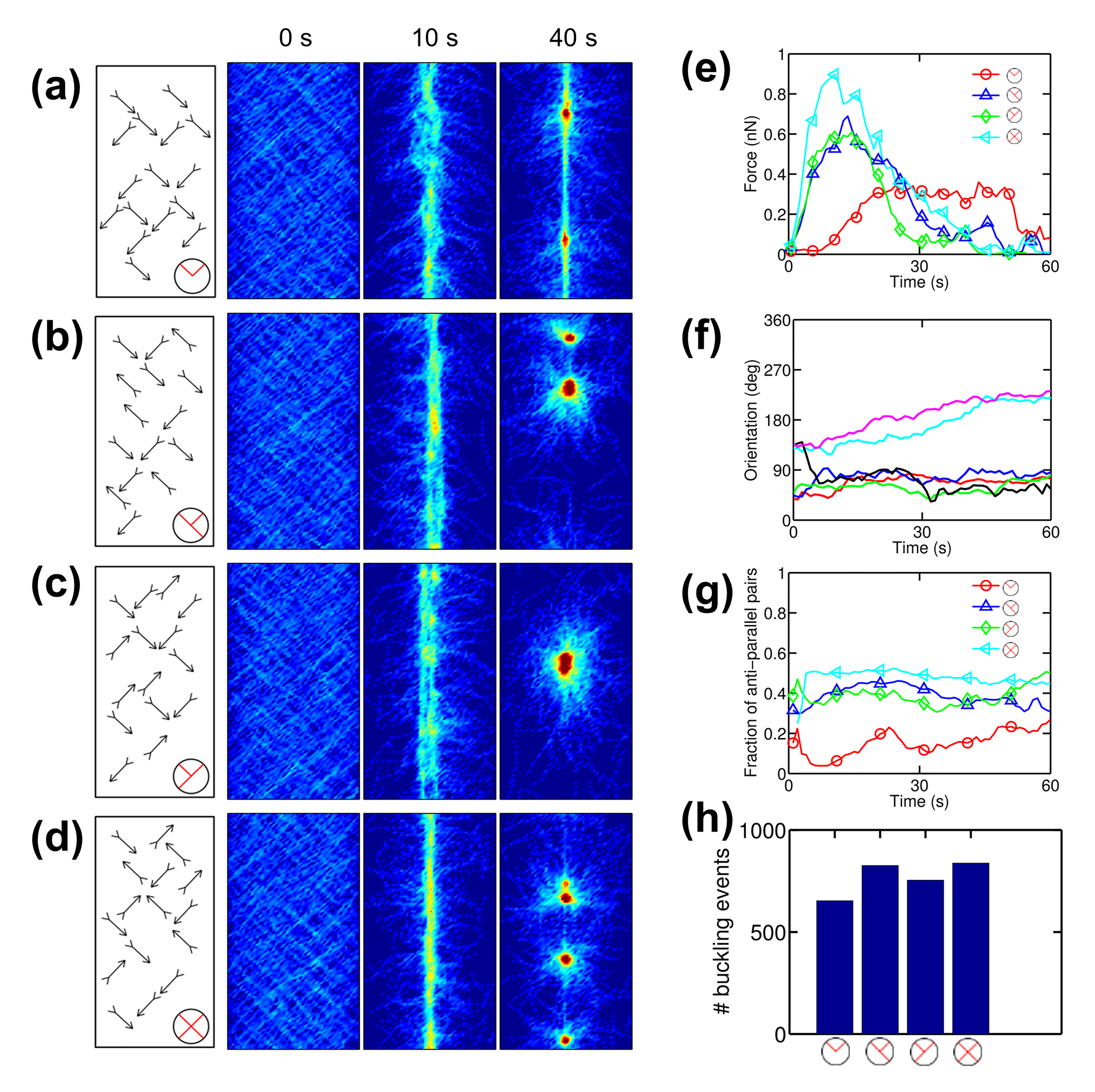

Supplement: S6 Fig — Densities of motors and ACPs used in cases shown here are 0.08 and 0.01, respectively. (a-d) (1st column) Orientations where barbed ends of actin filaments in networks are initially directed. Red lines on the circles located at the bottom-right corner represent the orientations. Arrows in the boxes represent examples of filaments with corresponding initial orientations. (2nd, 3rd, 4th columns) Snapshots showing actin density in the networks at t = 0, 10, and 40 s with initial orientation indicated in the 1st column. (e) Time evolution of tension for cases shown in (a-d). (f) Time evolution of orientations of selected actin filaments in the case shown in (a). (g) Time evolution of a fraction of antiparallel filament pairs for cases shown in (a-d). (h) Number of buckling events occurring during simulations for cases shown in (a-d). (TIF) [file pcbi.1005277.s008.TIF]

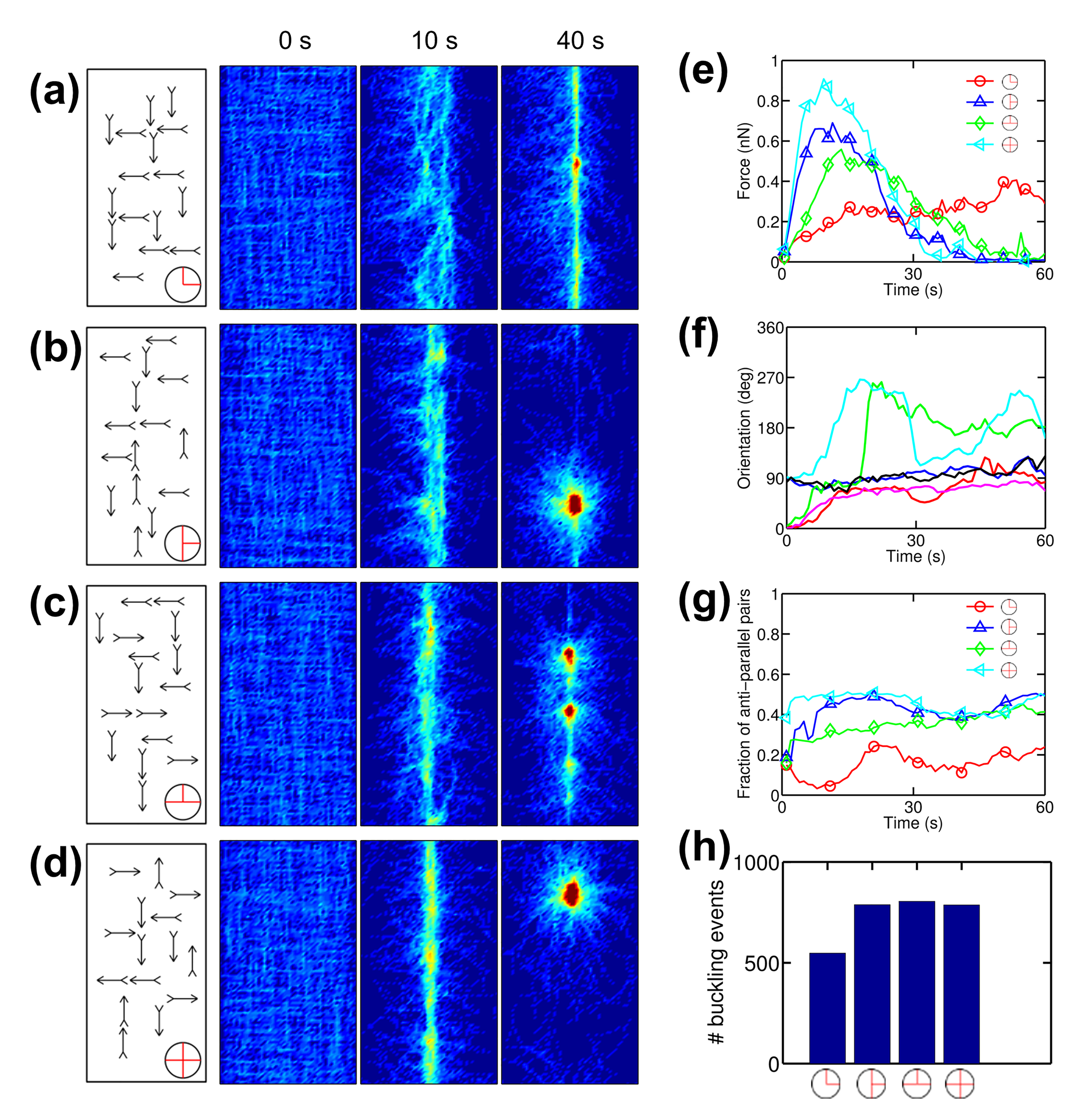

Supplement: S7 Fig — Densities of motors and ACPs used in cases shown here are 0.08 and 0.01, respectively. (a-d) (1st column) Orientations where barbed ends of actin filaments in networks are initially directed. (2nd, 3rd, 4th columns) Snapshots showing actin density in the networks at t = 0, 10, and 40 s with initial orientation indicated in the 1st column. (e) Time evolution of tension for cases shown in (a-d). (f) Time evolution of orientation of selected actin filaments from case shown in (a). (g) Time evolution of a fraction of antiparallel filament pairs for cases shown in (a-d). (h) Number of buckling events occurring during simulations for cases shown in (a-d). (TIF) [file pcbi.1005277.s009.TIF]

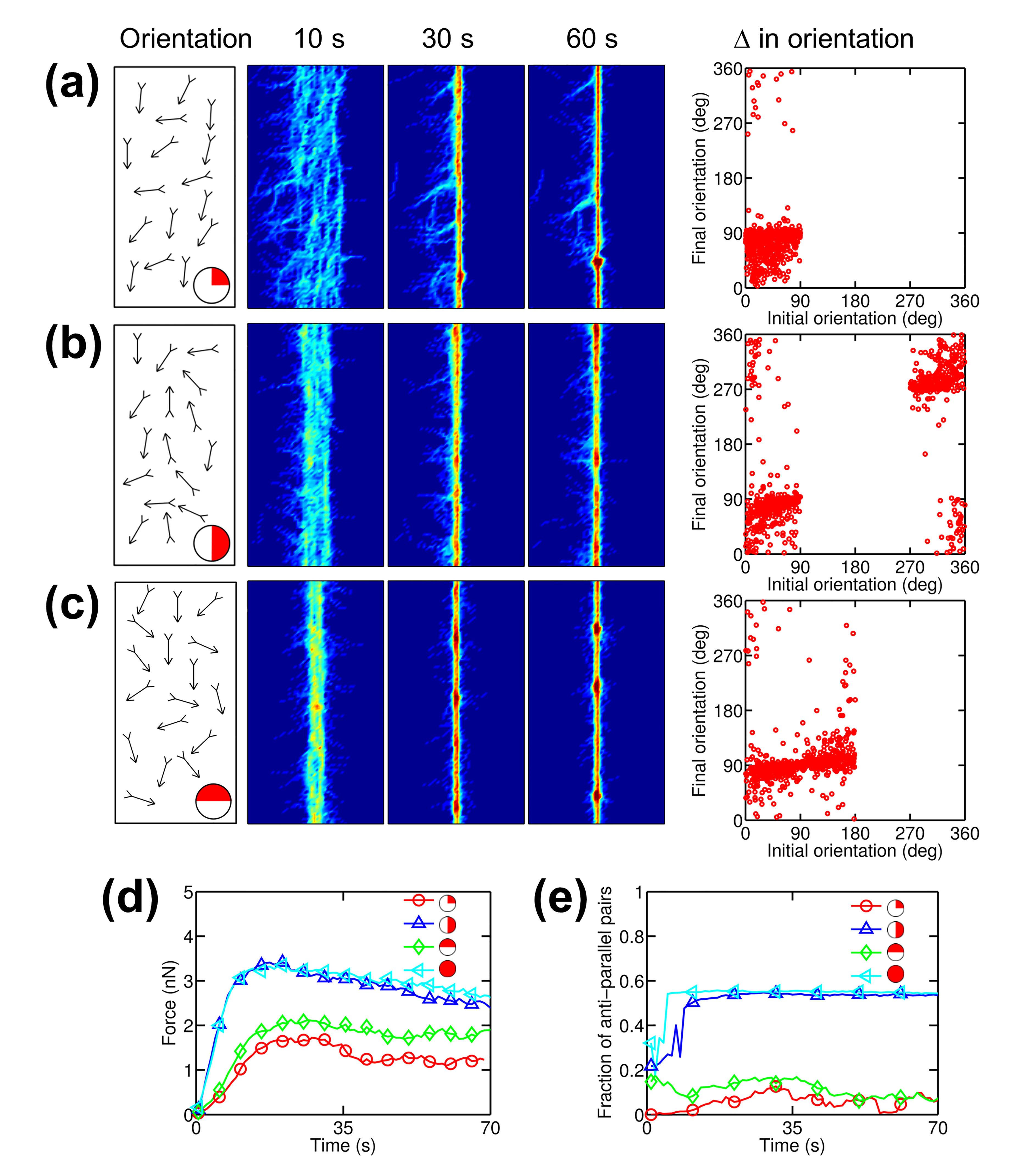

Supplement: S8 Fig — Densities of motors and ACPs used in cases shown here are 0.08 and 0.1, respectively. (a-c) (1st column) Orientations where barbed ends of actin filaments in networks are initially directed. (2nd, 3rd, 4th columns) Snapshots showing actin density in the networks at t = 10, 30, and 60 s with initial orientation indicated in the 1st column. (5th column) Initial and final orientations of actin filaments. Final orientation indicates orientation of filaments measured at a time point when compaction time is defined. (d) Time evolution of tension for cases with biased initial orientations shown in (a-c) and isotropic initial orientation. (e) Time evolution of a fraction of antiparallel filament pairs for cases shown in (d). (TIF) [file pcbi.1005277.s010.TIF]

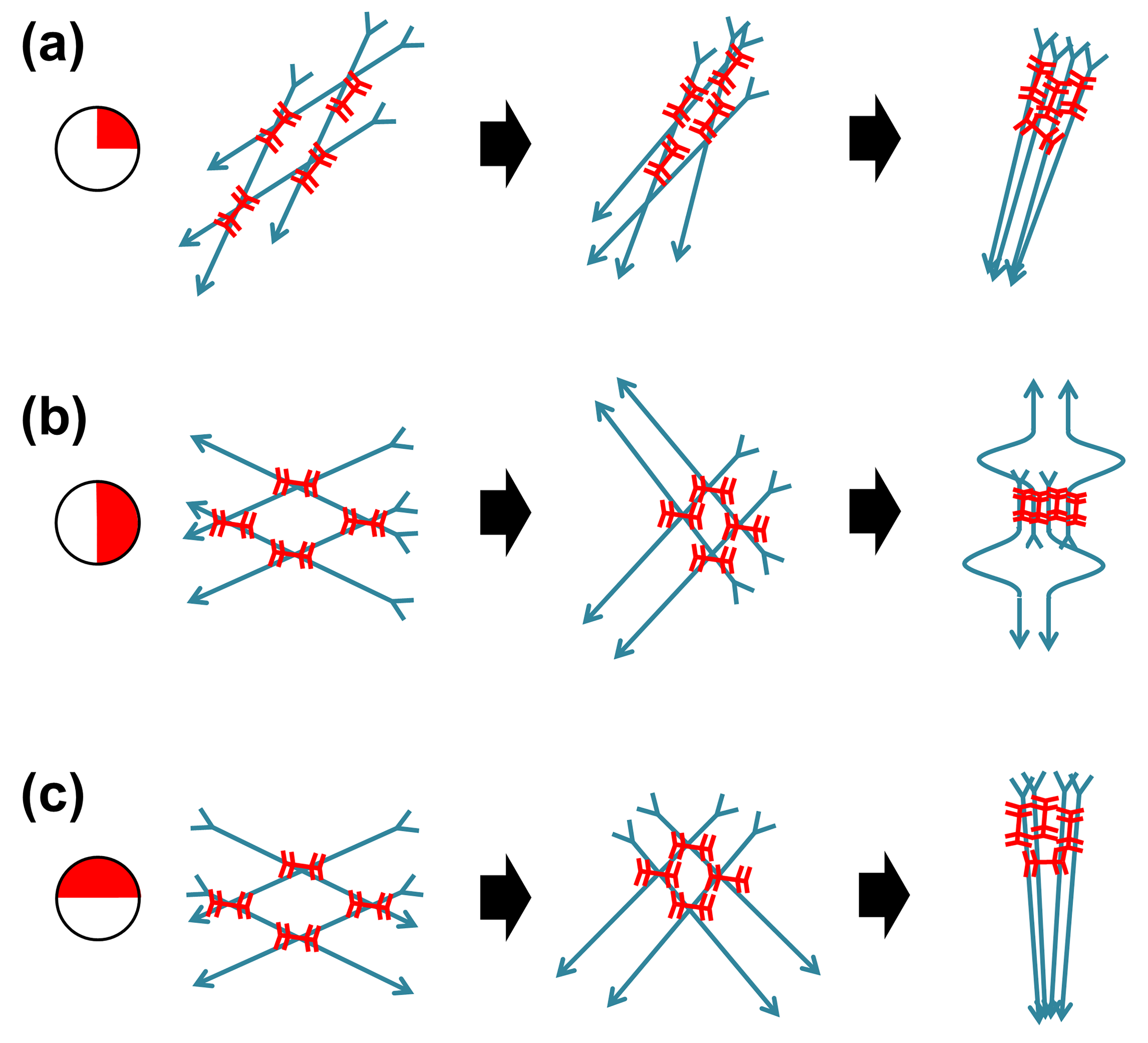

Supplement: S9 Fig — Schematic diagrams show actin filaments and motors initially directed toward (a) +x/+y, (b) +x/±y, and (c) ±x/+y as in cases shown in Fig 4A–4C. Teal and red represent actin filaments and motors, respectively, whereas ACPs are not shown for simplicity. Because the periodic boundary condition exists only in the y-direction, actin filaments tend to be aligned in the y-direction. (a) Most of the actin filaments oriented toward +x/+y are aligned in parallel via polarity sorting. (b) Antiparallel pairs of actin filaments initially oriented relatively in the y-direction can be aligned well in the y-direction. However, the alignment results in the buildup of compressive forces on the actin filaments unlike in other cases. If bending stiffness of actin filaments is low enough, the actin filaments are buckled and oriented in the y-direction. If buckling is suppressed due to large bending stiffness, the actin filaments cannot be oriented in the y-direction. (c) Antiparallel pairs of actin filaments initially oriented relatively in the x-direction can be aligned in the y-direction via polarity sorting. (TIF) [file pcbi.1005277.s011.TIF]

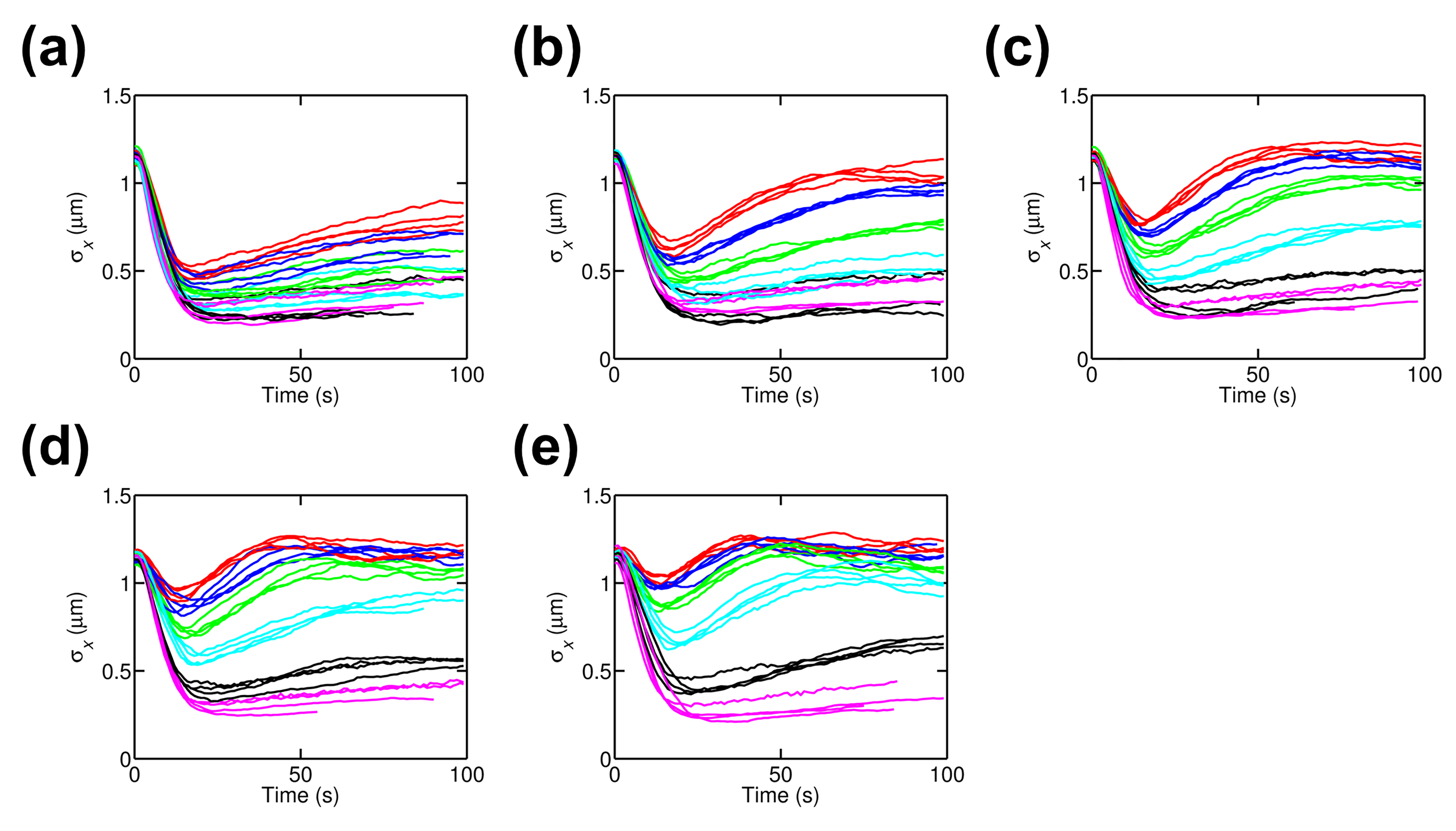

Supplement: S10 Fig — The turnover rate used in these cases is (a) 30 s-1, (b) 45 s-1, (c) 60 s-1, (d) 75 s-1, and (e) 90 s-1. The inhibition factor is 0 (red), 0.2 (blue), 0.4 (green), 0.6 (cyan), 0.8 (black), and 1 (magenta). (TIF) [file pcbi.1005277.s012.TIF]

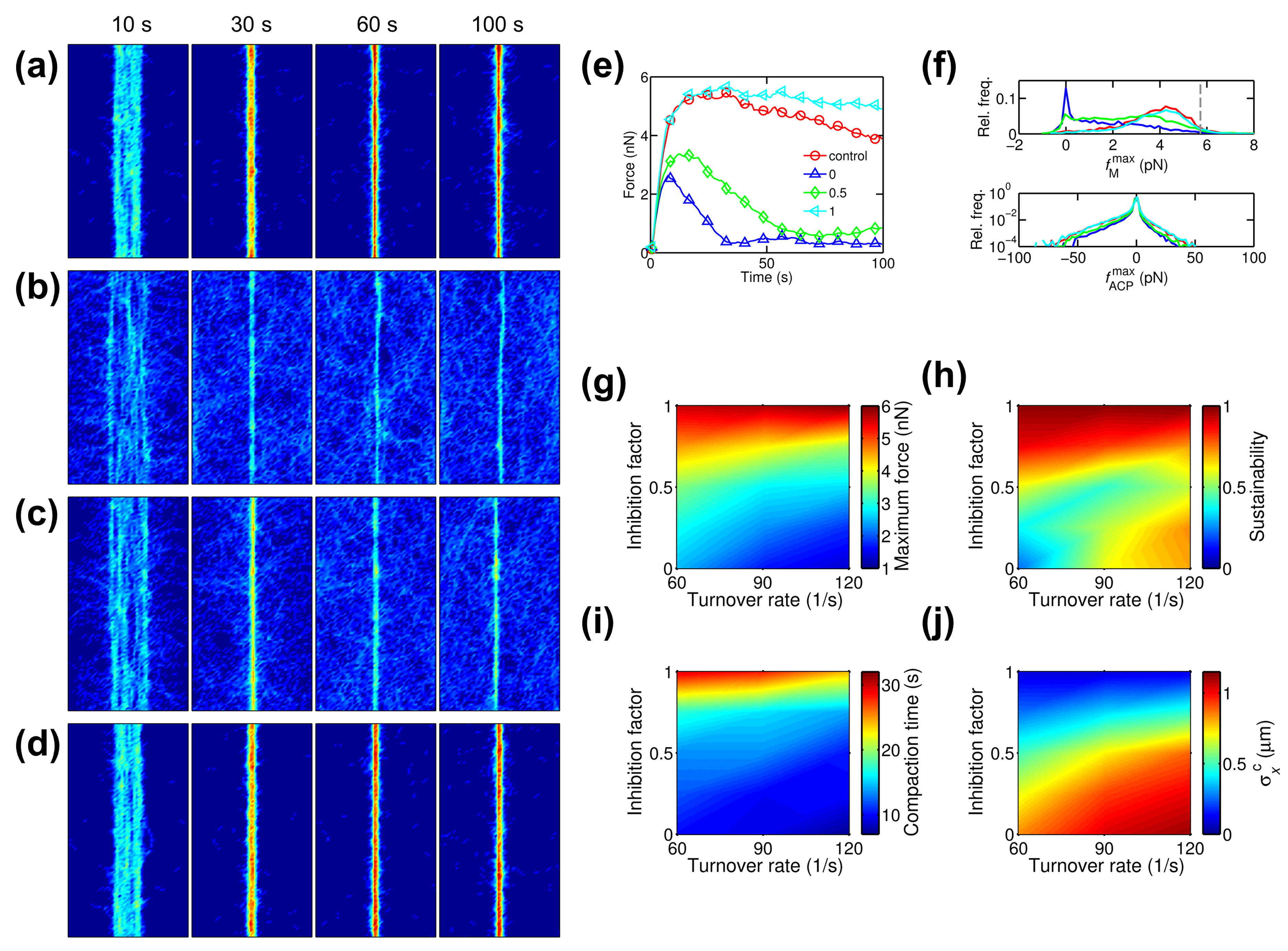

Supplement: S11 Fig — Densities of motors and ACPs used in cases shown here are 0.08 and 0.1, respectively. (a-d) Snapshots showing actin density in networks (a) without actin turnover and (b-d) with actin turnover rate (kt,A) of 60 s-1. In networks with actin turnover, depolymerization of actin filaments was inhibited by bound ACPs or motors to an extent determined by inhibition factor (ξd,A). ξd,A ranges between 0 (no inhibition of depolymerization) and 1 (complete inhibition). In these examples, ξd,A is (b) 0, (c) 0.5, or (d) 1. (e) Time evolution of tensile forces generated by bundles for cases shown in (a-d). (f) Distribution of forces exerted on motors (fMmax) and ACPs (fACPmax) measured at peak tension for cases shown in (a-d). The gray dashed line indicates stall force of motors (5.7pN). The legend is shared with (e). (g) The maximum and (h) sustainability of tension, depending on kt,A and ξd,A. (i) Compaction time. (j) Standard deviation of x positions of actins at the compaction time (σxc). (TIF) [file pcbi.1005277.s013.TIF]

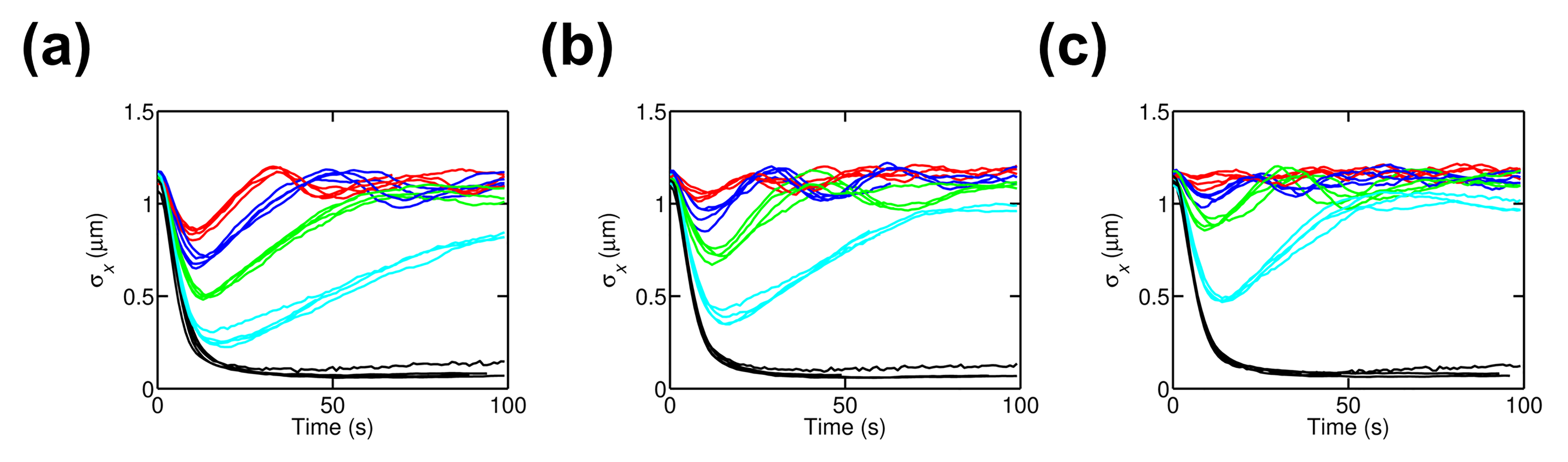

Supplement: S12 Fig — The turnover rate used in these cases is (a) 60 s-1, (b) 90 s-1, and (c) 120 s-1. The inhibition factor is 0 (red), 0.25 (blue), 0.5 (green), 0.75 (cyan), and 1 (black). (TIF) [file pcbi.1005277.s014.TIF]
